# Differential Regulation of Breast Cancer-Associated Genes by Progesterone Receptor Isoforms PRA and PRB in a New Bi-Inducible Breast Cancer Cell Line

**DOI:** 10.1371/journal.pone.0045993

**Published:** 2012-09-24

**Authors:** Junaid A. Khan, Catherine Bellance, Anne Guiochon-Mantel, Marc Lombès, Hugues Loosfelt

**Affiliations:** 1 Institut National de la Santé et de la Recherche Médicale (INSERM) Unité 693, Steroid Receptors: Endocrine and Metabolic Pathophysiology, Le Kremlin-Bicêtre, France; 2 Université Paris-Sud, Faculté de Médecine Paris-Sud, Unité Mixte de Recherche UMR S693, Le Kremlin-Bicêtre, France; 3 Assistance Publique-Hôpitaux de Paris, Hôpital Bicêtre, Service de Génétique moléculaire, Pharmacogénétique et Hormonologie, Le Kremlin-Bicêtre, France; 4 Assistance Publique-Hôpitaux de Paris, Hôpital Bicêtre, Service d’Endocrinologie et Maladies de la Reproduction, Le Kremlin-Bicêtre, France; 5 Department of Physiology and Pharmacology, University of Agriculture, Faisalabad, Faisalabad, Pakistan; Institut de Génomique Fonctionnelle de Lyon, France

## Abstract

Progesterone receptor isoforms (PRA and PRB) are expressed at equal levels in normal mammary cells. However, alteration in PRA/PRB expression is often observed in aggressive breast cancer suggesting differential contribution of PR isoforms in carcinogenesis. The mechanisms underlying such processes remain to be established mainly due to paucity of appropriate cellular models. To investigate the role of PR isoforms and the impact of imbalanced PRA/PRB ratio in transcriptional regulation, we have generated an original human breast cancer cell line conditionally expressing PRA and/or PRB in dose-dependence of non-steroid inducers. We first focused on PR-dependent transcriptional regulation of the paracrine growth factor gene amphiregulin (*AREG)* playing important role in cancer. Interestingly, unliganded PRA increases *AREG* expression, independently of estrogen receptor, yet inhibitable by antiprogestins. We show that functional outcome of epidermal growth factor (EGF) on such regulation is highly dependent on PRA/PRB ratio. Using this valuable model, genome-wide transcriptomic studies allowed us to determine the differential effects of PRA and PRB as a function of hormonal status. We identified a large number of novel PR-regulated genes notably implicated in breast cancer and metastasis and demonstrated that imbalanced PRA/PRB ratio strongly impact their expression predicting poor outcome in breast cancer. In sum, our unique cell-based system strongly suggests that PRA/PRB ratio is a critical determinant of PR target gene selectivity and responses to hormonal/growth factor stimuli. These findings provide molecular support for the aggressive phenotype of breast cancers with impaired expression of PRA or PRB.

## Introduction

Progesterone receptor (PR) is a ligand-induced transcription factor belonging to the nuclear receptor family of steroid hormone receptors [1]. PR exists as two isoforms, PRB and PRA, transcribed by alternate initiation of transcription from two distinct estrogen-regulated promoters [2]. PRB is a full length 114 kDa protein, comprising of 933 amino acids while PRA is truncated in the N-terminal region (94 kDa) and lacks the first 164 amino acids. Despite the high sequence similarity, accumulating evidence indicates that PRB and PRA are functionally distinct transcriptional factors [3] and exhibit differential physiological responses in target tissues [4]–[6]. Biochemical and biophysical studies suggest that unique conformation of PRA and PRB [7] allows interaction with distinct coregulators [8]. Under physiological conditions, the majority of PR positive mammary epithelial cells express both PR isoforms at equimolar levels [9], [10]. However, altered PRA/PRB ratio is often associated with breast carcinogenesis [9], [11], [12]. Several lines of evidence indicate that PRA/PRB ratio greatly influences breast cancer outcomes. Metastasis associated protein 1 overexpression in transgenic mice led to tumorigenesis concomitant to elevated PRA/PRB ratio [13]. Genetic predisposition to cancer development due to mutations in *BRCA1* or *BRCA2* leads to PRA overexpression that may play a role in disease progression [14], [15]. Likewise, in endometrial cancers, disrupted PRA/PRB expression is observed and cancers with elevated PRA/PRB ratio are also correlated with poor prognosis [16]. Moreover, transgenic mice overexpressing PRA exhibit abnormal mammary gland development characterized by extensive lateral branching, ductal hyperplasia and disorganized basement membrane with decreased cell to cell adhesion [17]. *In vivo* relationship between PRA/PRB ratio and antiprogestin responsiveness revealed that decreased PRA/PRB ratio is associated with antiprogestin resistance [18]. Furthermore, restoration of balanced PRA/PRB ratio using methyltransferase inhibitors re-established antiprogestin sensitivity in mouse mammary carcinomas [19] suggesting that PR isoforms differentially contribute towards cancer progression.

Recent studies show that PR may by-pass progesterone (P4) requirement for transcriptional activation of certain gene subsets [20], [21] and sexual behavior induced by dopamine in mice involves unoccupied PR [22]. Therefore, it seems plausible to define PR as a sensitive transcription factor capable of responding not only to its cognate ligand but also to diverse cellular stimuli/growth factors irrespective of hormonal status. Given that several aspects of progesterone (P4) signaling are differentially influenced by PR isoforms, PRA/PRB ratio should be considered as an important determinant of functional consequences of P4 signaling. We have recently reported a major role of p38 and p42/44 mitogen-activated protein kinases (MAPKs) in regulating PRA/PRB expression ratio at post-translational level [23] that might influence P4 signaling in PR expressing cells. Most of previous studies [24]–[26] on transcriptional regulation by PR isoforms were conducted in T47D cells (ER+) expressing either PRA or PRB where PR homodimers are the only molecular species or in separate cell lines expressing each PR isoform. Although these studies provided insights in transcriptional regulation by PRs, however, genetic background related effects coming from separate cell lines expressing PRA or PRB makes the comparison difficult for distinct PR isoforms transcriptional properties. Given that three molecular species (PRA, PRB homodimers, PRA-PRB heterodimers), with likely different functions, may exist in PR positive cells, changes in PRA/PRB ratio should profoundly influence signaling of ligand-free as well as liganded PR [27].

To better understand genomic effects of varied PRA/PRB expression, we have generated a bi-inducible breast cancer cellular model allowing controllable PRA/PRB ratio. Using this model, we show that functional outcome of epidermal growth factor (EGF) and P4 signaling is dependent on PRA/PRB ratio as well as on target gene promoter context. Furthermore, PR isoforms distinctly impact the transcriptional regulation of heparin-binding growth factor (*HBEGF*) and amphiregulin (*AREG*), paracrine growth factors implicated in carcinogenesis. We demonstrate that unliganded PRA continuously increases *AREG* transcription, independently of estrogen receptor, that can be inhibited by antiprogestin RU486. Therefore, increased *AREG* expression by elevated PRA/PRB ratio may provide paracrine growth stimulus for proliferation of nearby PR negative cells. By extension to these studies, we established the transcriptomic profiles of the bi-inducible cell line identifying distinct gene subsets regulated by ligand-free and hormone-bound PRA and PRB. We show that PR-responsive transcription is highly influenced not only by the presence of hormone but also by selective PR isoform expression. Moreover, our studies highlight that imbalanced PRA/PRB ratio may increase the impact of progestins on breast cancer development and metastases. This newly established cellular model thus constitutes a powerful tool to discriminate the differential impact of PR isoforms in cancer progression.

## Results

### Establishment and Characterization of Stable Cell Line Expressing Inducible PR Isoforms

**Figure 1:**
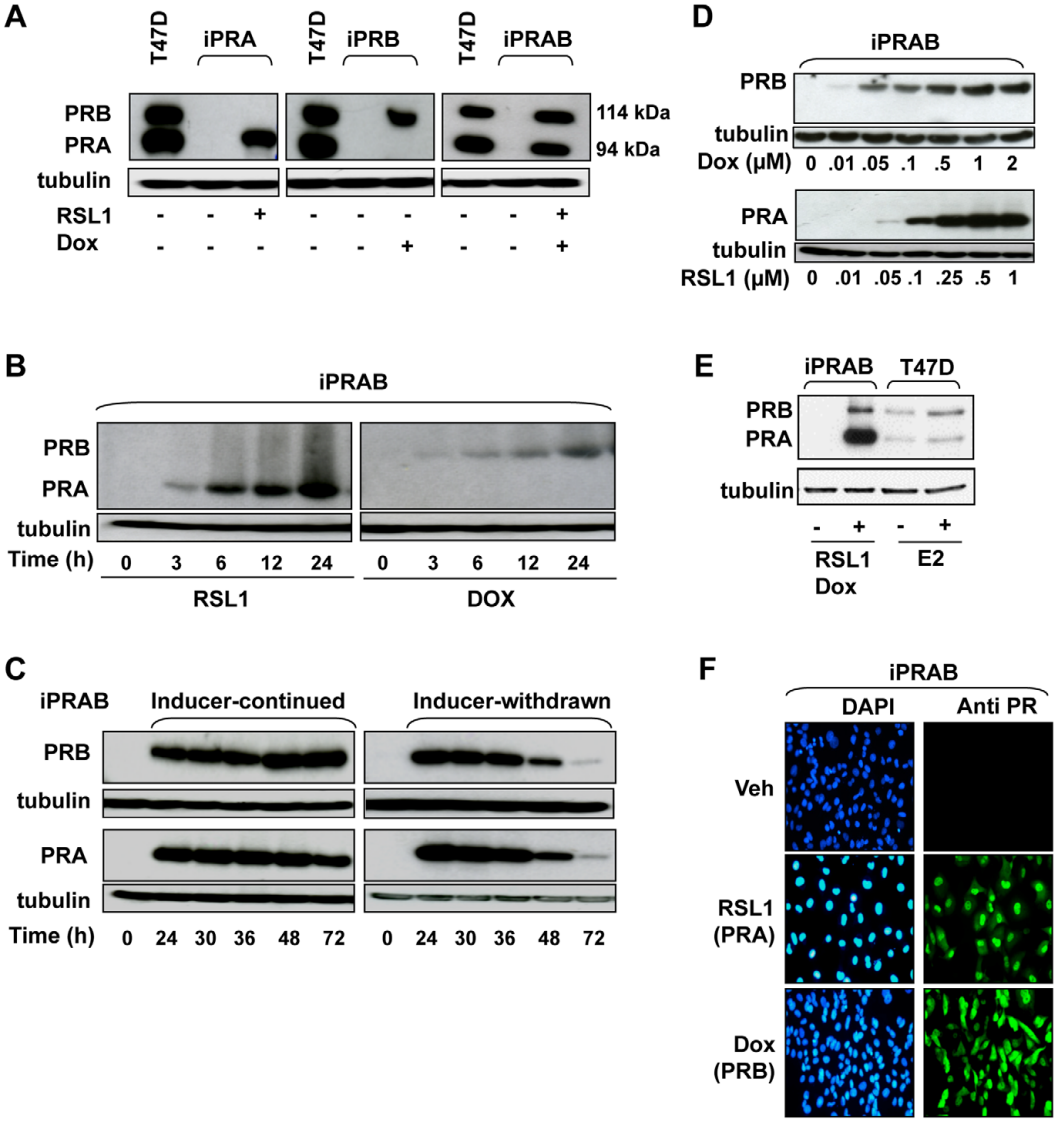
Inducible expression of PRA and/or PRB in iPRA, iPRB or iPRAB cell lines. (**A**) The iPRA or iPRB or iPRAB cells were incubated or not with inducer(s), RheoSwitch Ligand (RSL1) (0.25 µM) and/or doxycycline (Dox, 1 µM) during 24 h and immunoblot analysis of whole cell extracts was performed using anti-PR antibody recognizing both PR isoforms (Novocastra) or anti-tubulin antibody for sample loading control. Inducible PRA and/or PRB electrophoretic bands were compared with endogenously expressed PR isoforms in wild-type T47D cells. (**B**) Cells were incubated with RSL1 (0.5 µM) or Dox (2 µM) during indicated time intervals and whole cell extracts were immunoblotted using antibodies against PR or loading control tubulin. (**C**) Cells were incubated with RSL1 (0.5 µM) or Dox (2 µM) during the indicated time intervals (left panels) or after 24 h of inducer exposure (right panels). Cells in the right panels were then rinsed twice with PBS and further incubated until the indicated times with fresh medium without inducer. Both series of cell extracts were immunoblotted as in A. (**D**) Cells were incubated with increasing concentrations of RSL1 or Dox as indicated for 24 h and immunoblot detection was performed as in A. (**E**) The iPRAB cells were incubated with RSL1 (1 µM) and Dox (1 µM) during 24 h and PR isoforms expression was compared with T47D cells treated or not with estradiol (E2, 10 nM) during 24 h. (**F**) The iPRAB cells were incubated with DMSO vehicle or RSL1 (0.5 µM) or Dox (2 µM) during 24 h and immunocytochemical analysis for PR isoforms detection was performed using Novocastra anti-PR antibody as described in *Materials and Methods*.

We used MDA-MB-231 cells (ER−, PR−) to establish three cell lines, conditionally expressing PRA (iPRA) or PRB (iPRB) or both PRA and PRB (iPRAB) using RheoSwitch and T-Rex systems which require the non-steroidal inducer ligands RheoSwitch Ligand (RSL1) and doxycycline (Dox) respectively. The regulatory proteins required for functioning of both of these systems were combined into the same plasmid pZX-TR including the zeocin resistance gene (Supplementary Fig. S1). Such combination was necessary to allow coordinated and balanced expression of the regulatory factors required for a bi-inducible system. Cells were stably transfected with pZX-TR plasmid and were screened for functioning of both inducible systems as described in Material and Methods. A representative clone (clone 250) was then stably transfected with pGALp-PRA and/or pTO-PRB expression vectors leading to generate iPRA, iPRB and iPRAB cell lines. Western blot analysis of the representative clone used for this study (iPRAB-38) together with iPRA and iPRB complementary clones only expressing one isoform is presented in Fig. 1A. For each of them, PR isoform expression can be tightly controlled by the respective inducer with undetectable PR expression in uninduced cells. Moreover, the PRA and PRB expression levels obtained at maximal induction of iPRAB cells were comparable to T47D cells endogenously expressing both PR isoforms (Fig. 1A).

One of the objectives of establishing bi-inducible cell line (iPRAB) was to precisely control the amount of each PR isoform in the same cells to allow the adjustment of PRA/PRB expression ratio at will. Time course experiments showed that PR expression was detectable as early as 3 h and increased continuously thereafter reaching a peak at 24 h (Fig. 1B) and maintained for up to 72 h (Fig. 1C, left panel) following single exposure of RSL1 or Dox. Removal of inducer after 24 h led to gradual decrease in PR isoform expression (Fig. 1C, right panel). This indicated that PRA and/or PRB expression could be easily switch-on or -off when desired. In addition, PR isoform expression levels could be adjusted by altering inducer concentrations. PRA levels were detectable from 0.05 µM RSL1 and reached a plateau at 0.5–1 µM concentration (Fig. 1D lower panel). Similarly, PRB expression was induced by Dox in the range of 0.01 to 1 µM (Fig. 1D upper panel). To highlight the flexibility of our system, we also compared the extent of PRA/PRB ratio in iPRAB cells with E2-inducible expression of PRA and PRB through constant ratio in T47D cells (Fig. 1E). Ligand binding assays allowed us to determine that, in the absence of inducers, expression leakage remained at low level (14 fmol/mg prot) as compared to P4 binding sites present in PR(−) MDA-MB-231 parental cells (7 fmol/mg prot). Following exposure to RSL1 (1 µM) or Dox (1 µM) for 24 h, PRA and PRB expression were induced up to 319 and 187 fmol/mg prot respectively. This should be compared to wild type T47D tumor cells expressing similar levels of PRA and PRB (around 900 fmol/mg prot) [28]. Thus, the iPRAB cell line allows us to adjust PRA/PRB ratio within the range 0.04–45, to be compared to the range 0–100 found in breast tumors [11]. Stability of the bi-inducible system was verified by immunocytochemical analysis of iPRAB-38 cells showing that more than 99% cells remained able to conditionally express either PRA or PRB in the presence of respective inducer(s) after 22 passages (Fig. 1F). As assessed by western blot analysis (Supplementary Fig. S2), the amplitude of induction of both expression systems was preserved after 22 passages upon RSL1 and/or Dox exposure without any detectable leakage in PR isoform expression in the absence of inducer(s).

Collectively, these results demonstrate that PR isoform expression levels in iPRAB-38 cell line can be tightly controlled by the amount of inducer(s), without genomic instability of the bi-inducible system. In subsequent studies, we focused on iPRAB-38 cells for determining the functional relevance of the system.

### Functional Integrity of Inducibly Expressed PR Isoforms in iPRAB Cell Line

Wild type PRs undergo progestin-induced turnover concomitant to ligand-induced PR phosphorylation at serine 294 [29] playing critical roles in PR signaling [30]. Therefore, we first analyzed whether inducible PR isoforms are capable of responding to such events. As shown in Supplementary Fig. S3, the inducibly expressed PRs underwent agonist-induced down regulation as well as antagonist-induced stabilization (Fig. S3A). In addition, PR isoforms were polyphosphorylated as shown by the electrophoretic upshift signature induced by P4 (Fig. S3B), and were phosphorylated at S294-PRB and S130-PRA key residues by both agonist (P4) and antagonist (RU486) ligands (Fig. S3C). Such properties were consistent with previous reports [23], [31], [32], and showed that the mechanisms of PR turnover/stabilization were functional in iPRAB cell line.

**Figure 2:**
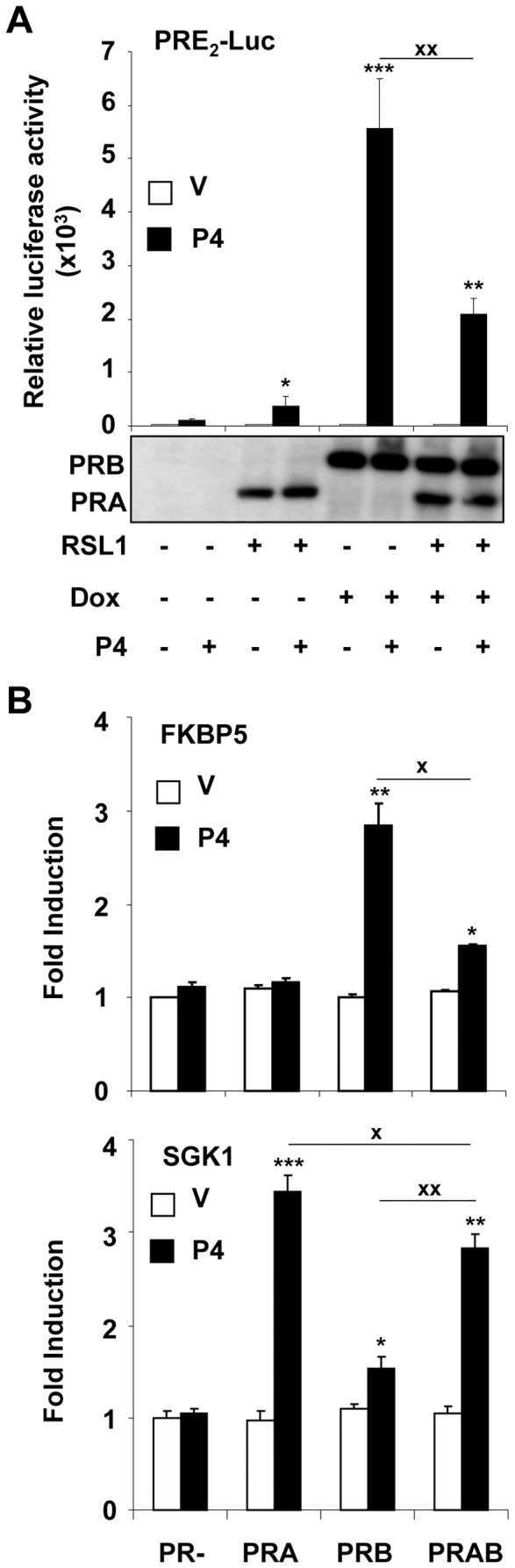
Inducibly expressed PR isoforms are functional by regulating reporter and endogenous gene transcription. (**A**) Following 24 h induction of PRA and/or PRB using RSL1 (0.5 µM) and/or Dox (2 µM) in steroid-free medium, iPRAB cells were transiently co-transfected with vector encoding PRE_2_-luciferase reporter gene and internal standard βGal expression vector as described in *Materials and Methods*. Following 24 h of transfection, cells were treated with vehicle (ethanol) or 10 nM progesterone (P4) during 6 h in steroid free medium. Cells were then rinsed twice with PBS, luciferase activity was determined and results (mean ± SEM) from six independent cell cultures are normalized to protein content (graphs). Statistical tests on difference either with PR− cells treated by vehicle (*) or between the indicated groups (x) are presented as described in *Material and Methods*. Western blot analysis of whole cell extract showing PR isoforms expression under similar induction and treatment conditions is presented (lower panel). (**B**) The iPRAB cells were induced as in A and then treated with vehicle (ethanol) or P4 (10 nM) during 6 h in steroid-free medium and quantitative real-time RT-PCR analysis was performed for determining *FKBP5* or *SGK1* transcripts levels as described in *Materials and Methods*. Data (mean ± SEM) from three independent cell cultures measured in duplicate are presented as fold increase in transcript levels as compared to vehicle-treated uninduced (PR−) cells. Statistical differences are presented as in A.

It has been reported that PRA and PRB regulate transcription to different extent from exogenous promoters comprising of consensus PRE sequences where PRA acts as a transrepressor of PRB activity, mainly due to the formation of less transcriptionally active PRA/PRB heterodimers [27], [33], [34]. To control the functional integrity of such functions in iPRAB cell line, we compared the effect of a given PRA/PRB ratio on several prototypal PR target genes. By using a transfected PRE_2_-luciferase reporter gene, we first calibrated the induction conditions leading to significant transrepression of PRB-dependent regulation for a PRA/PRB ratio of 0.5 ((Fig. 2A lower panel). In uninduced iPRAB cells, transactivation by P4 was negligible confirming the low background expression of PR isoforms in the absence of inducer(s) (Fig. 2A). As expected, co-expression of both PRA and PRB (Fig. 2A lower panel) strongly decreased luciferase activity as compared to PRB alone, while the intrinsic activity of PRA was very low (Fig. 2A upper panel). Such transcriptional profiles supported that PRA and PRB were functional in iPRAB cells. We then examined the impact of the same PRA/PRB ratio on endogenous transcription of FK506 binding protein 5 (*FKBP5*) and serum and glucocorticoid-regulated kinase 1 (*SGK1*) genes known to be directly regulated by PR (Fig. 2B). P4 induced transcription of *FKBP5* specifically by PRB and co-expression of PRA led to decreased PRB transcriptional response in a similar way as the transrepressive effect of PRA on PRB-mediated transcription from exogenous promoter (Fig. 2A). Interestingly, although PRA was expressed at lower level than PRB, it behaves as a much stronger transcriptional factor as compared to PRB on *SGK1* gene (Fig. 2B, lower panel). In such context of PR isoform opposed activities, co-expression of both isoforms resulted in intermediate levels of *SGK1* transcripts indicating that PRB can also decrease PRA-mediated transcription in contrast to the classical model. These results support that the activity of one isoform may be compromised when the other isoform is co-expressed. Conversely, from the PRAB condition, suppression of one isoform expression may result in inverted switches on distinct target genes, suggesting that imbalance of PRA/PRB ratio may differentially affect transcription of genes sensitive to both isoforms. These experiments confirmed that inducible PR isoforms were functional in iPRAB cell line and differentially acted on endogenous transcription allowing us to investigate their impact on cancer-related gene expression.

### PR Isoforms Differentially Contribute to Antiproliferative Effects of Antiprogestin RU486

**Figure 3:**
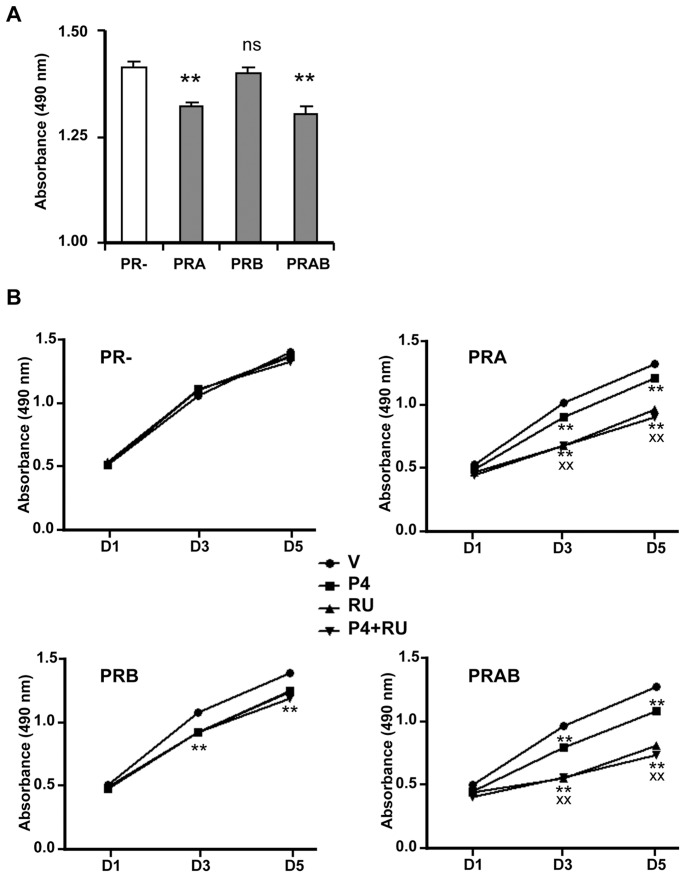
Antiproliferative efficacy of antiprogestin RU486 depends on PRA or PRB isoform expression. (**A**) Approximately 5,000 iPRAB cells per well were cultured in 96-well plates in steroid-free medium during 24 h. On Day 0, 2 and 4, fresh steroid-free medium was replaced containing or not RSL1 (0.5 µM) and/or Dox (2 µM) to respectively induce expression of either PRA or PRB or both (PRAB) or none of them (PR−). Cell proliferation assays were performed using CellTiter 96® AqueousOne Solution as described in *Materials and Methods* and absorbance at 490 nm, representative of total number of living cells was determined at the indicated time (days). Data (mean ± SEM) from six independent cell cultures (n = 6) are presented. Stars (*) represent statistical difference between uninduced (PR−) and PR isoforms(s) expressing cells in the absence of ligand. (**B**) PR isoforms(s) expression was induced as above then cells were treated by vehicle or P4 (10^−8^ M) or RU486 (10^−6^ M). Cell proliferation was determined on day 1, 3 and 5 as in A. Data (mean ± SEM) from six independent cell cultures (n = 6) are presented, the low error bars being masked by point symbols (maximal SEM for the four graphs was 0.028 for PRA under P4 at day 5). Statistical differences at p<0.01 with either vehicle (**) or P4 alone (xx) -treated cells are indicated and were calculated by Mann-Whitney test. For clarity, only one statistical test is presented for the closest or overlapped points giving similar p-values.

P4 exerts either growth stimulatory or inhibitory effects depending on target cells [35]–[39]. We thus examined the relative contribution of PR isoforms on cell proliferation in the presence of vehicle or P4 and/or antiprogestin RU486. The proliferation of cells expressing ligand-free PRA was significantly decreased as compared to PRB-expressing cells and co-expressing PRB did not modify the antiproliferative effect of unliganded PRA (Fig. 3A). In uninduced (PR−) cells, neither P4 nor RU486 modified cell proliferation (Fig. 3B) whereas both P4 and RU486 exerted antiproliferative effects when either or both PR isoforms were expressed. Interestingly, while P4 exerted similar effects via PRA or PRB, the antiproliferative efficacy of RU486 was stronger in PRA and PRA+PRB induced cells as compared to PRB-expressing cells. These results indicate that the antiproliferative responses of P4 or RU486 in iPRAB cells are specifically mediated via PR and importantly, the antiprogestin exerts differential antiproliferative efficacy depending on selective PR isoforms expression.

### *HBEGF* and *AREG* Transcription is Differentially Influenced by Ligand, Growth Factors and PR Isoforms

**Figure 4:**
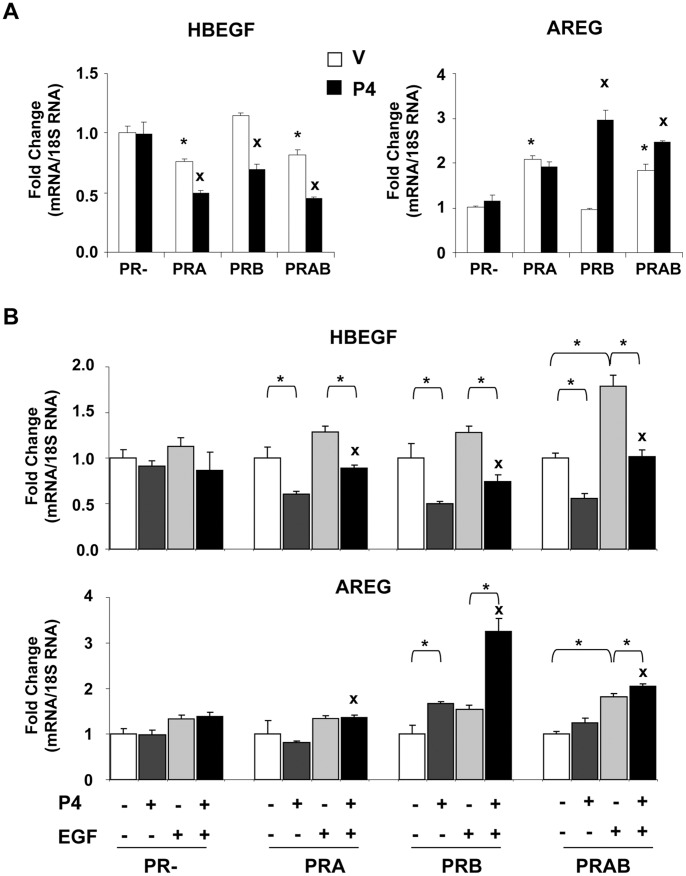
PR isoforms distinctly regulate transcription of *HBEGF* and *AREG* genes. (**A**) Following 24 h induction of PRA and/or PRB expression using RSL1 (0.5 µM) and/or Dox (2 µM) in steroid-free medium, cells were treated with vehicle or P4 (10 nM) during 6 h and qRT-PCR analysis was performed for determining *HBEGF* and *AREG* mRNA levels as described in *Materials and Methods*. Data (mean ± SEM) from three independent cell cultures measured in duplicate are presented as fold change in transcript levels as compared to that in vehicle-treated uninduced (PR−) cells. Statistical difference is indicated as compared to vehicle condition for either uninduced (PR−) cells (*) or the corresponding PR-induced cells (x). (**B**) Cells were incubated with RSL1 (0.25 µM) and/or Dox (1 µM) during 24 h in steroid-free medium and then treated with vehicle or P4 (10 nM) or EGF (30 ng/ml) or both during 6 h. qRT-PCR analysis was performed as above and data (mean ± SEM) are presented as fold change with respect to the corresponding induced vehicle-treated cells. Statistical difference is indicated either as star (*) for the indicated treatment groups or as cross (x) for P4+EGF *vs* P4-treated cells.

Recent *in vivo* studies have shown that long term P4 treatment elicits proliferation of PR(−) mammary cells by paracrine mechanism whereas PR(+) cells remain insensitive to such effects [40], [41]. Heparin-binding epidermal growth factor (*HBEGF*) and amphiregulin (*AREG*) are highly implicated in mammary gland carcinogenesis and metastasis [42], [43] and are both regulated by PR [44]. We analyzed their differential regulation by inducible PR isoforms. Quantitative RT-PCR analyses from iPRAB cell line (Fig. 4A) showed that while *HBEGF* transcription was significantly down-regulated by unliganded PRA and PRA-PRB, P4 exposure decreased *HBEGF* expression under all PR isoforms conditions. In contrast, PRA increased *AREG* transcript levels irrespective of hormone (Fig. 4A) whereas PRB up-regulated *AREG* transcription in P4-dependent manner. Co-expression of PRA and PRB mixed both effects. We excluded a potential artifact of RSL1 (Supplementary Fig. S4), and confirmed that unliganded PRA increases *AREG* mRNA expression in other cell models as well as amphiregulin synthesis (Supplementary Fig. S5). These results indicated that elevated PRA/PRB ratio alone has a significant impact on *AREG* expression.

EGF signaling interferes with PR-regulated pathways at multiple levels including interactions of PR with MAPK cascades [45], increased transcriptional activity of PRB [46] and PR isoform stability/turnover [23]. Therefore, we explored the impact of EGF on PR isoform-mediated transcriptional regulation of *HBEGF* and *AREG* genes. PRA and/or PRB expressions were decreased by reducing inducer concentrations to minimize the possible saturating effects on transcription. EGF did not modify *HBEGF* expression in the absence of PR (Fig. 4B, upper panel) while it increased *HBEGF* transcript levels, although non-significantly, in the presence of unliganded PRA or PRB. The latter effect became significant when both isoforms were co-expressed. This shows that EGF-dependent up-regulation of *HBEGF* requires both PRA and PRB highlighting the importance of growth factor stimuli in eliciting transcriptional responses of PR isoforms even in the absence of hormone. While P4 treatment decreased *HBEGF* expression irrespective to PR isoforms as expected from Fig. 4A, surprisingly, P4 counteracted EGF-stimulated *HBEGF* expression when either or both PR isoforms were expressed indicating that EGF and P4 exert opposite effects on *HBEGF* transcription via PR. EGF differently impacted the transcriptional regulation of *AREG* (Fig. 4B, lower panel). As shown in Fig. 4B, EGF alone slightly increased *AREG* transcription independently of PR. As expected, decreasing PR expression led to lowered P4-induced PRB-mediated *AREG* expression as compared to Fig. 4A. Interestingly, simultaneous treatment with P4 and EGF led to synergetic increase in *AREG* transcription in PRB- but not in PRA-expressing cells, highlighting the important role of EGF signaling in governing PR transcriptional properties. However, this effect was compromised by co-expression of PRA. This indicates that selective PR isoform expression strongly influences synergetic responses to EGF and P4 on *AREG* transcription. Collectively, our data underscore the pivotal role of P4 and EGF signaling cross-talk as well as the impact of PRA/PRB ratio for controlling key target genes such as *HBEGF* and *AREG* involved in breast cancer development and metastasis.

### Unliganded PRA Increases *AREG* mRNA Levels at Transcriptional Level

**Figure 5:**
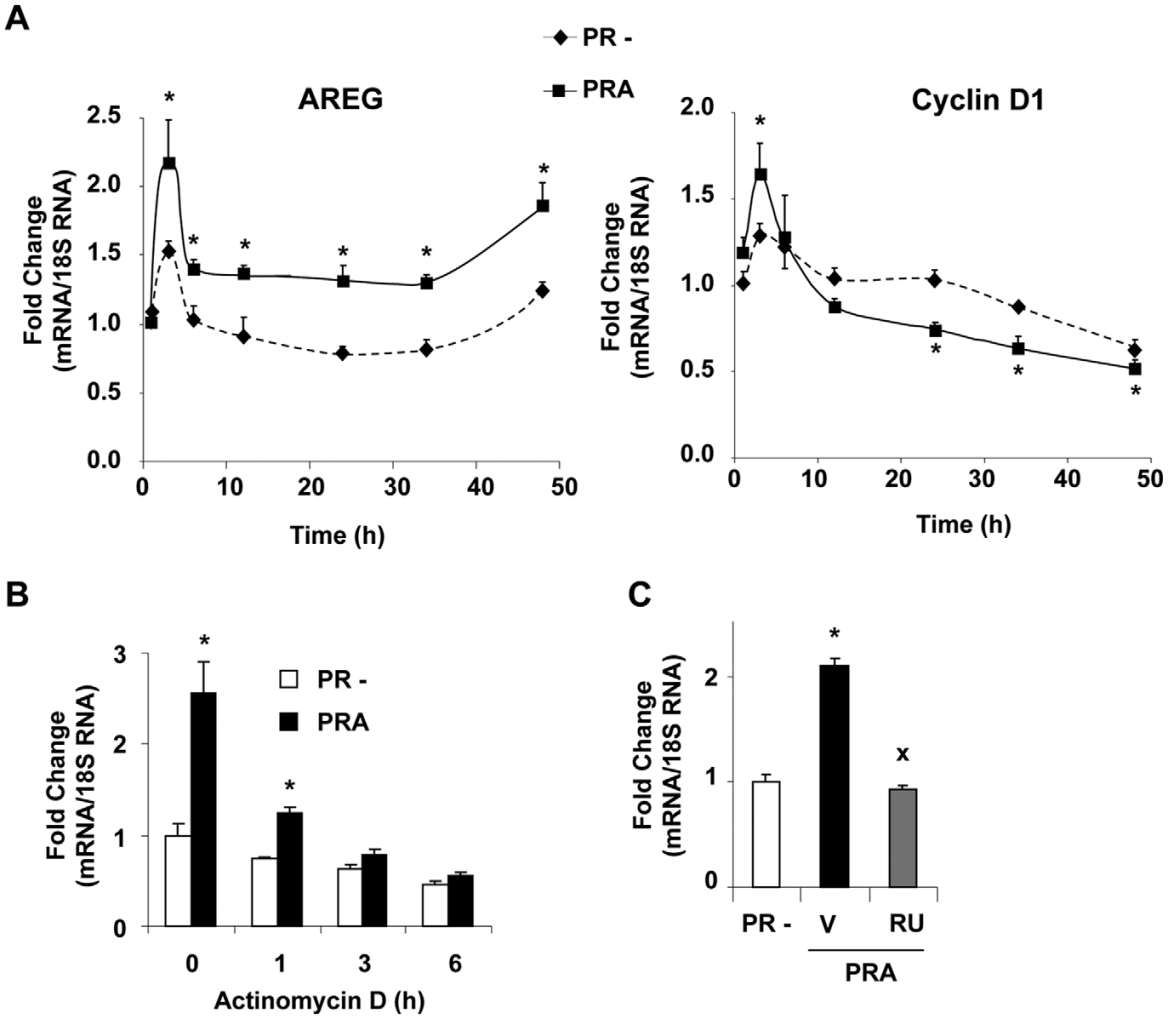
Unliganded PRA induces continuously elevated *AREG* mRNA levels. (**A**) Cells were incubated with DMSO vehicle or RSL1 (0.5 µM) in steroid-free medium for indicated time periods and qRT-PCR analysis was performed for *AREG* or cyclin D1 transcript levels as indicated. Data (mean ± SEM) from three independent cell cultures measured in duplicate are presented as fold change with respect to transcript levels at 1 h time period in uninduced cells. Star (*) represents statistical difference with uninduced (PR−) cells for the corresponding time point. (**B**) Following 24 h exposure to DMSO vehicle or RSL1 (0.5 µM), cells were incubated with actinomycin D (4 µM) during indicated time periods and qRT-PCR analysis of AREG mRNA expression was performed. Star (*) represents statistical difference with uninduced (PR−) cells for the corresponding time point. (**C**) Cells were induced or not for 24 h as in B and then treated with vehicle or antiprogestin RU486 (10 nM) during 6 h. AREG mRNA was quantified by qRT-PCR and data (mean ± SEM) from three independent cell cultures in duplicate are presented as fold change with respect to vehicle-treated uninduced cells (PR−). Statistical differences with either uninduced PR− cells (*) or vehicle-treated PRA-induced cells (x).

Owing to the functional significance of *AREG* as a paracrine growth factor in cell proliferation, we examined the kinetics of *AREG* expression along with cell cycle regulatory gene cyclin D1, following PRA induction. As shown in Fig. 5A, concomitantly to the initial boost likely due to renewal of culture medium, *AREG* mRNA levels increased as early as 3 h after PRA expression, and remained elevated up to 48 h as compared to *AREG* levels in uninduced PR(−) cells, indicating that PRA induces continuous expression of this paracrine factor. Under similar conditions, cyclin D1 transcript levels significantly increased following 3 h of PRA expression and then continuously decreased as compared to PR negative cells consistent with a sustained antiproliferative effect of unliganded PRA (see Fig. 3A). This suggests that PRA-expressing cells might be insensitive to continuously increased *AREG* expression, which nevertheless might influence proliferation of nearby PR negative cells through its well known paracrine growth stimulatory effects.

We next examined the molecular mechanisms by which unliganded PRA increased *AREG* mRNA levels. PRA expression was induced during 24 h and cells were then incubated with actinomycin D, an inhibitor of transcription, during 1 to 6 h. As shown in Fig. 5B, elevated *AREG* levels in PRA-expressing cells gradually decreased following actinomycin D treatment and reached similar levels as in uninduced cells providing strong evidence that unliganded PRA regulates *AREG* expression at transcriptional level. We finally demonstrated that antiprogestin RU486 completely blocked PRA-mediated increase in *AREG* transcription (Fig. 5C), confirming the role of unliganded PRA in *AREG* transcriptional activation. Taken together, these results indicate that elevated PRA/PRB ratio increases *AREG* expression irrespective of P4 and can be controlled by PR antagonist RU486. Up-regulation of such paracrine growth factors by unliganded PRA might contribute towards aggressive phenotype of PRA-dominant breast tumors. This raised the question of how PRA and PRB isoforms could impact the whole cancer-related genes in iPRAB cell line.

### PRA/PRB Expression Determines PR-target Gene Selectivity Depending on Ligand Status

**Figure 6:**
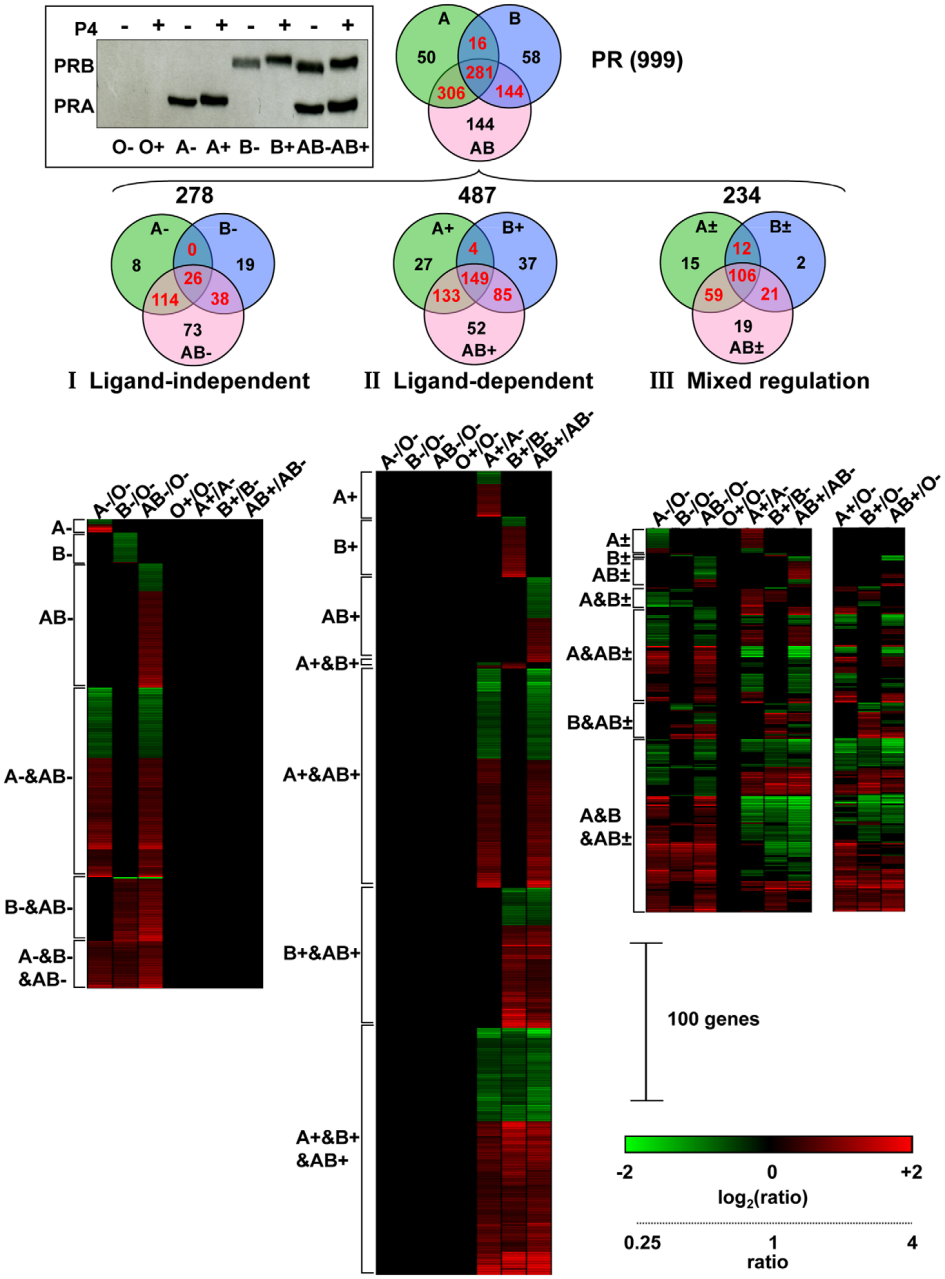
Gene expression profiles following PR isoform conditional expression and hormonal treatment. The iPRAB cells were treated for 24 h by RSL1 (0.25 µM) and/or Dox (1 µM) to induce expression of either PRA (A) or PRB (B) or PRA plus PRB (AB) expression and then incubated with vehicle (−) or 10^−8^ M progesterone (+) for 6 h. Control cells were not treated by inducers (O). PR isoforms expression was analyzed by western-blot (upper left inset). PRA/PRB ratio value corresponding to each condition was estimated according to ligand binding assays and western blot quantification as described in *Material and Methods* (A: 45; B: 0.04; AB: 1). The corresponding total RNAs (O−, O+, A−, A+, B−, B+, AB−, AB+) were extracted and gene expression profiling was done using Agilent 44K-oligonucleotide microarrays as described in *Materials and Methods*. Venn diagrams showing the total number of genes regulated for each subset and clustering analyses (heat maps) of differentially expressed genes in each experimental condition were performed as described in *Materials and Methods*. Each conditional expression as compared to the reference RNA is indicated on the top of heat maps. The gene clusters corresponding to Venn diagram areas are indicated on the left side of heat maps (−: ligand-independent; + ligand-dependent; ±: mixed regulation; &: intersection). As indicated in the lower right panel, color intensities reflect the log_2_ (ratio) values obtained for down-regulated (green) and up-regulated (red) genes, and were saturated at log_2_ (ratio) of ±2 corresponding to FC ±4.

In order to identify the potential candidates for PR-dependent carcinogenesis and to gain insights on the global changes in gene expression by hormone-free as well as liganded PR isoforms, we performed genome-wide transcriptomic studies in iPRAB cell line. Following 24 h induction of either PRA or PRB or PRA + PRB, cells were treated with vehicle or P4 for 6 h and RNA expression was profiled using gene expression chips relevant for the whole human transcriptome (Fig. 6). A total of 999 genes (including 953 protein-coding genes) were directly or indirectly affected by PR expression. These genes were primarily distributed into three partially overlapped groups A, B, and AB irrespective to ligand condition (Fig. 6, upper Venn diagram). Group AB includes genes impacted by PR isoforms co-expression at similar level and is thus representative for the transcriptional pattern of PR-regulated genes in the usual physiological conditions. The group A or B includes genes regulated by PRA or PRB respectively and corresponds to the new transcriptional patterns observed in cell lacking the other isoform as compared to the AB condition. Although previous data obtained in various conditions of PR expression, ligand and cell lines from microarray studies are not directly comparable, 172 genes matching with our list were at least once reported to be regulated by PR and are listed in Supplementary Table S2. In contrast, our findings are relevant for the intrinsic properties of PR isoforms expressed in a single cell line. Furthermore, we identified 827 new genes impacted directly or indirectly by PR expression among which many exert major functions. Complete list of genes along with the fold changes (FC) and p-values in expression levels corresponding to hormonal condition and PR isoform expression is provided in Supplementary Table S3.

In order to compare all transcriptional profiles obtained for the seven conditional expressions for a given gene, we applied a cut-off at FC ±1.3 and p-value 10^−3^. This allowed us to define two classes of genes exclusively responding to PR isoforms either in a ligand-independent (class I) or ligand-dependent (class II) mechanism. All other genes variably responding to both conditions were classified in a mixed regulation category (class III). Of note, for the three classes of genes, the amplitudes of transcriptional responses to either PRA or PRB or both of them were frequently similar in sharp contrast to transcription previously obtained with a PRE_2_ synthetic reporter gene (see Fig. 2A), indicating that PRA can act as strongly as PRB on endogenous transcription invalidating the notion that PRA is a weak transcriptional factor.

Mapping of PR isoform specificities are represented as Venn diagrams associated with the corresponding heat maps in Fig. 6 quantifying the distinct as well as common genes regulated by unliganded and liganded PR isoforms in down (green) or up (red) direction. The detailed description of each gene is given in Supplementary Table S3 according to the heat map order shown in Fig. 6. Class I genes regulated in the absence of hormone were not further impacted by the addition of P4 and are thus ligand-independent genes (Fig. 6, left panel). Unliganded PRA and PRB regulated the transcription of common but also distinct gene populations. Co-expressed PRA and PRB regulated the dominant gene population in the same direction (60% up- vs 40% down-regulated), highly suggesting that PRA and PRB transcriptionally cooperate under hormone-free conditions through unknown mechanism. It should be noted that, in the absence of hormone, the dominant gene subset (114 genes, 62% up- *vs* 38% down-regulated) lied in the intersection between A and AB groups (A−&AB−) reflecting that transcription of such genes requires PRA but can be modulated or not by PRB co-expression. Therefore, such genes are PRA dependent and might be sensitive or insensitive to PRB co-expression. In contrast, while almost all genes exclusively responding to unliganded PRB were down-regulated (B− subset), genes lying in intersection B−&AB− were up-regulated (37 among 38 genes). This suggests that PRA may be required to trigger PRB up-regulating activity independently of hormone on certain promoters. In the presence of P4 (Fig. 6, middle panel), a non-overlapped population of genes was selected that did not respond to unliganded PR isoforms but only to liganded species (class II). Of note, the proportion of genes down-regulated by hormone (about 40%) was comparable to the fraction up-regulated, showing that progestin-induced transconformation of PR leads to transcriptional repression as well as activation mainly depending on promoter context. In this second class, the dominant gene subset (149 genes) was commonly regulated by PRA, PRB and PRA-PRB (A+&B+&AB+ subset), and, here again, a major gene subset (133 genes) was PRA dependent (A+&AB+). Such genes are responsive to liganded PRA and their expression may or may not be influenced by PRB co-expression. Similarly, PRB-dependent gene subset (B+&AB+) may or may not be modulated by PRA co-expression (85 genes). Interestingly, in such intersected populations of genes, including the A+&B+&AB+ subset, we did not identify genes regulated in opposite directions by the corresponding conditional expressions. Moreover, only 4 hormone-responsive genes were sensitive to either PRA or PRB but insensitive to isoform co-expression (A+&B+). This has to be compared with the absence of class I genes commonly regulated by unliganded PRA or PRB but not by co-expressed isoforms (A−&B−). Therefore these results highlight the dominant role of PRA and PRB cooperativity in regulating transcription. For both class I and II, exclusive genes for PRA or PRB were also found that only respond to one isoform expressed alone (8 genes A−, 27 genes A+, 19 genes B−, 37 genes B+) i.e. are shifted to basal level by co-expression of the other isoform irrespective to their activation or repression. These genes are thus likely controlled by isoform-specific transrepression mechanism irrespective to their up or down direction, and are specifically triggered by imbalanced PRA/PRB ratio. Genes responding only when PRA and PRB are equally co-expressed constituted the major gene population (73 genes AB− vs 52 genes AB+). Importantly, these genes are shifted to the basal level by the lack of one isoform. Genes AB+ are likely to be highly sensitive to P4-dependent A/B heterodimers. In contrast, since PRA and PRB have not been shown to heterodimerize in the absence of ligand, the AB− genes (Fig. 6 left panel) are likely regulated by distinct mechanism involving promoter-dependent cooperativity of both isoform monomers. While the majority of genes specifically responding either to the absence or the presence of hormone (class I and II), a large population (class III, 234 genes) was also sensitive to both conditions in a differential manner (Fig. 6, right panel). In order to compare class III transcriptional patterns to the same reference O−, we transformed the ratios A+/A−, B+/B− and AB+/AB− to A+/O−, B+/O− and AB+/O− respectively (Fig. 6 additional right panel). This allows visualizing gene clusters regulated by PR isoform(s) in opposite directions depending on hormonal status (including *ADAM12, ADAMTS6, ESM1, IL1B, SERPINB2*). Moreover, some of these clusters revealed that the presence of P4 can switch the up or down expression of genes regulated by unliganded PR back to the basal level. Finally, the transcriptional variations of *AREG* (A±&B±&AB±), *FKBP5* (B+), *HBEGF* (A±&B±&AB±), *and SGK1* (A+&AB+) genes obtained from our microarray studies as for other genes tested (not shown) highly correlated with those previously measured by real time RT-qPCR.

Next, in order to discriminate the transrepressive functions from the cooperative effects of both co-expressed isoforms, we calculated the ratios of A *vs* AB and B *vs* AB signatures respective to each condition of ligand, giving the new heat map patterns additively shown in Supplementary Table S3. Such calculated ratio allowed comparing in the same cells the effect of a lack in PRA or PRB expression as compared to equal expression of PRA and PRB. Surprisingly, in contrast to the usual model, a majority of genes moved by lack of PRA were down-regulated independently of ligand (68%) or under the control of P4 (56%), while lack of PRB led to similar proportion of inhibiting effects on distinct or common genes (71% *vs* 60%). This new labeling of genes might be thus relevant for the biological processes affected by imbalanced PRA/PRB expression ratio usually found in PR+ cancer cells.

Together these results provide evidence that PR-dependent gene expression profile not only depends on the presence or absence of hormone but importantly also on the relative expression of individual PR isoform. Any strong deregulation of PRA/PRB ratio would thus lead to a significant shift not only in transcriptional amplitude for a given gene but also in selection of distinct gene subsets resulting in a highly flexible transcriptional fingerprint.

### Impact of PR Isoform Conditional Expression on Cancer-related Genes

**Figure 7:**
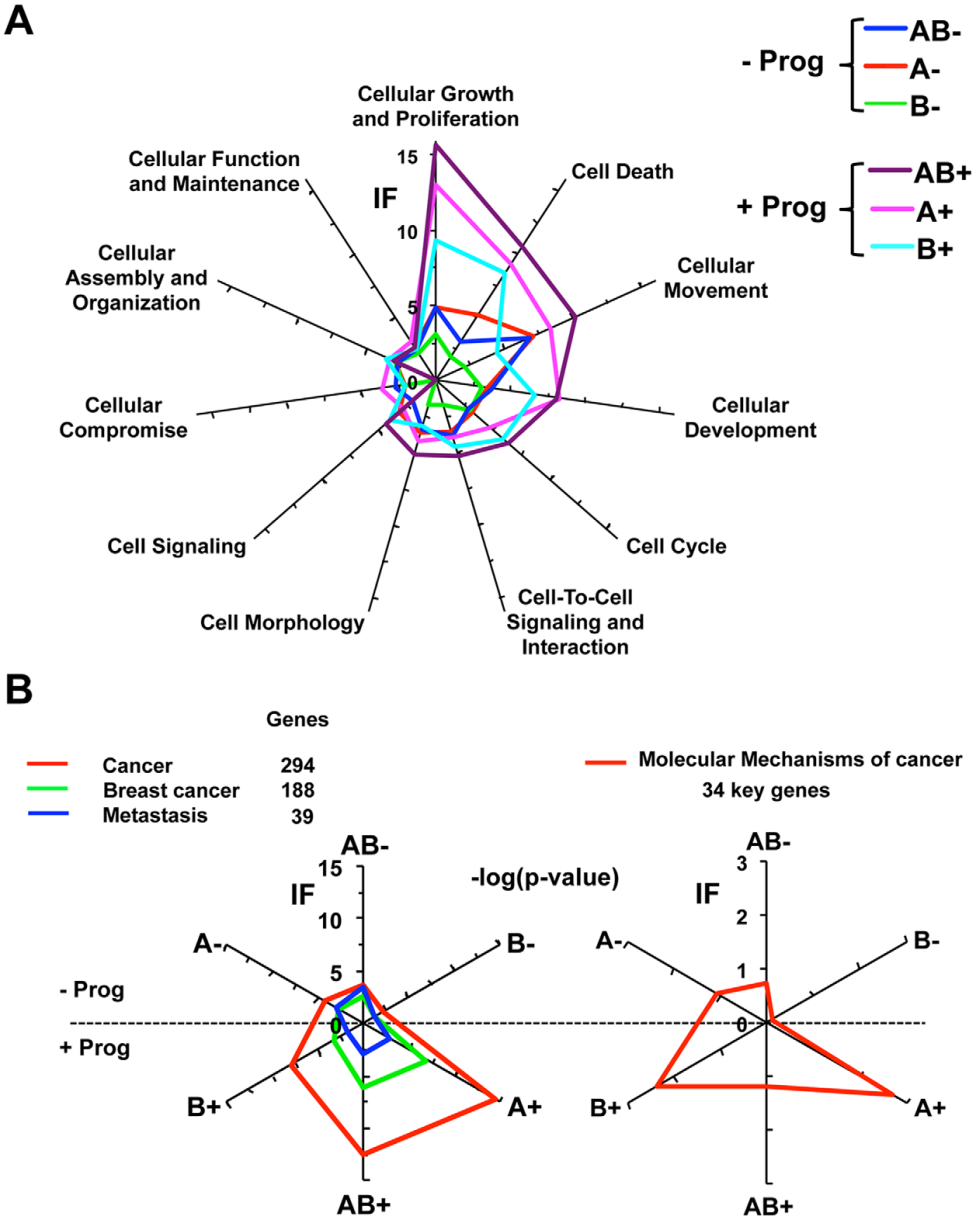
Functional impact of unliganded and liganded PR isoform(s) on cellular functions and cancer processes. Genes regulated by selective PR isoform(s) expression and hormonal conditions were analyzed using the Ingenuity Pathway Analysis system (IPA) as defined in *Material and Methods*. The transcriptional effects on biofunctions (cut-off FC ±1.3) for each conditional expression of PRA A), PRB (B) or PRA plus PRB (AB) in the absence (−) or presence (+) of P4 were evaluated by an impact factor (IF) corresponding to –log(IPA p-value). (**A**) The impact of isoforms on main cellular functions are represented by a polar chart where each curve corresponds to variation of IF for a given condition. (**B**) The impact on cancer, breast cancer, metastasis (left panel) and molecular mechanisms of cancer (right panel) is represented on a polar chart where each axis symbolizes the indicated condition, and each curve corresponds to variation of IF for a given disease. The vertical axis corresponds to equimolar expression of PRA and PRB (AB), while the oblique axes represent the unbalanced ratio conditions for PRA (A) or PRB (B). The hormonal status is symbolized at the opposite poles of each axis (− : no ligand.; +: P4). The corresponding number of genes is indicated.

We next employed both Ingenuity (IPA) and PANTHER classification System to investigate the key biological functions linked to the identified genes and classified them into functionally related subgroups. The complete gene ontology annotations obtained from both systems are given for each gene in Supplementary Table S4, giving the facility to sort any functional gene list using keywords to be compared with the fold changes heat maps. In addition, complete gene ontology classification obtained from IPA and PANTHER systems are given in Supplementary Table S5. From these data we extracted the heat maps shown in Supplementary Fig. S6. PR isoform conditional expression highly impacted various biological pathways involved in cancer signaling with a predominant role of PRA in various cytokine-dependent regulatory pathways. The impacts of such regulations on cellular functions is evaluated by the polar chart shown in Fig. 7A. The graph provides evidence for the predominant role played by hormone in all these functions and highlights that PRA impacts cell growth, cell death and cell migration processes more than PRB. Also in the absence of ligand, PRA is more effective than PRB in regulating cell migration and cell death processes.

We further determined the relative impact of each conditional expression on the most represented regulatory pathways and cellular processes into the whole PR-regulated functional pattern (Supplementary Fig. S7A and B). In the absence of ligand, PRB clearly lacks activity in both categories in contrast to unliganded PRA that significantly targets all the pathways and cellular functions examined with a predominant impact on cytokine and TGFβ signaling. Hormone addition triggers PRB activity predominantly on interleukin, EGF, semaphorin and PI3 kinase signalings, while it widely increases the field of action of PRA in a proportional manner without preferentially targeting a particular pathway or cellular function. Co-expression of PRA and PRB clearly results in selection of new transcriptional targets in a cooperating manner. This shows that, in terms of gene selectivity, PRA seems to be the functionally dominant isoform and does not act as a repressor of PRB on the main regulatory pathways and cellular functions.

Most of the PR-regulated functions described above are critical in cancer development. We thus estimated their impact in ‘diseases and disorders’ category from IPA knowledge database. The top category found reassembled genes involved in general cancer processes and is represented as a polar chart (Fig. 7B, left), together with gene subsets known to play a role in breast cancer and/or metastasis. This highlights that PRA (i.e. lack of PRB) displays the higher impact factor in all these contexts. In addition, focusing on key genes involved in molecular mechanisms of cancer (Fig. 7B, right), 34 genes were found to be differentially regulated by PR isoforms. Surprisingly, the impact of PRB (i.e. lack of PRA) was clearly enhanced in this category although PRA remained prominent. These results thus revealed that the lack of PRA as well as PRB expression might strongly enhance the impact of PR-regulated genes in carcinogenesis processes as compared to equal expression of both isoforms in PR+ cells.

We next merged a total of 200 genes from the lists of PR-regulated genes affecting breast cancer, metastasis and molecular mechanism of cancer, and sorted them in function of their PR-dependent down or up regulations potentially influencing tumor growth and metastasis (Supplementary Table S6). This list highlights that 75% of up-regulated and 25% of down-regulated cancer-related genes regulated by PR may affect tumor growth in unfavorable direction (90 genes). Genes regulated by unliganded PRA were again predominant as compared to unliganded PRB. P4 treatment further increased the number of PRA− as compared to PRB-regulated genes involved in such diseases. Co-expression of PRA and PRB gave almost similar results than those obtained by PRA alone suggesting that the major impact of P4 signaling on tumorigenesis might preferentially involve PRA isoform. We further calculated transcriptional ratios giving the relative effect of a lack of either PRA or PRB expression as compared to the PRA-PRB co-expression condition. The list of 96 differentially regulated genes shown in Table 1 indicates that PR-dependent transcriptional variations potentially impacting breast cancers in unfavorable direction is dramatically influenced by the fall in PRA or PRB expression. Although some genes were similarly affected by such defect irrespectively to the isoform (such as *DKK1*, *CXCL2*, *S100P*, *TGFB2, WNT11)*, the functional consequences of a defect in PRA expression were surprisingly predominant by either stimulating (*AREG*, *EREG, F3, FKBP5, FZD2*) or repressing (*BRCA2, CASP8, DACH1, FOXC1*) hormone-sensitive genes. Moreover, genes such as *BCL3, CXCL1* and *HBEGF* were impacted by the lack of PRA in a hormone insensitive manner. Similarly, the lack of PRB expression specifically induced transcriptional changes on key genes such as *AHR, BCL2A1, IL1B, JAK1* and *MMP14*.

**Table 1:** Impact of imbalanced PRA/PRB ratio on transcription of genes involved in breast cancer development and metastasis.

	−P4		+P4	
	Lack of PRB	Lack of PRA	Lack of PRB	Lack of PRA
Down	*BLID, BMPR2, JAK1,* *PPP2R5C, SMYD4,* *VHL*	*ADRB2, BRCA2, MITF, PPP2R5C,* *RAD50, RGS4, SERPINB2*,*SMYD4, VHL*	*BIK, BLID, CDKN1A, CEBPD,* *CTDSPL, FOXO1, HIC1, OXTR,* *PPP2R5C, RGS16, SOCS1,* *TACC2, TNFRSF10B, VHL*	*BIK, BRCA2, CASP8, CDKN1C, DACH1, DUSP6,* *FOXC1, HIC1, PPP2R5C, RAD50, RGS4,* *SERPINB2, SMYD4, TACC2, TFPI2,* *TNFRSF10B, VHL, ZFP36*
Up	*EGLN3, GJB2, IL1A,* *SSTR2*	*BCL3, BIRC3, CSF2, CXCL1,* *EDN1, FOSL1, HBEGF, ICAM1,* *ITGAV, JAG1, KIAA1199, MMP9,* *NFKB2, NFKBIE, PDGFRB, PITX2,* *RELB, RHOJ, RHOU, S100P,* *TGFB2, THBS1, TMSB4X, TNS4,* *WNT11*	*ADAMTS1, AHR, BCL2A1, CDK6,* *CSF2, CXCL2, DKK1, FOSL1,* *HDAC9, HELB, IER2, IL1B, IL8,* *INHBA, IRS1, ITGA2, KRT17,* *LETM2, MMP14, NR3C2, ODC1,* *PIK3R3, PITX2, PLK2, PRKAR2A,* *S100P, TGFB2, TM4SF1,* *TMSB4X, TNS4, WNT11*	*ADAM12, ADRA1B, AGPAT9, AREG, ATP6V0A4,* *BCL3, BIRC3, BMP1, CSF2, CXCL1, CXCL2,* *CXCR4, CYP1A1, CYP1B1, DDIT4, DKK1, EDN1,* *EREG, EZR, F3, FKBP5, FOSL1, FZD2, HBEGF,* *ICAM1, IER2, IL8, IMPDH1, IRS2, ITGA2, JUN,* *KIAA1199, LETM2, LPAR1, MLPH, MMP9,* *NFKB2, NFKBIE, PTGER4, RASGRF1, RASGRP1,* *RHOU, S100P, SCG5, SOX9, TFAP2C, TGFB2,* *THBS1, TMSB4X, TNS4, UGCG, ZNF367*

Transcriptional changes induced by lack of either PRA or PRB as compared to the pattern obtained in cells co-expressing PR isoforms at equimolar level and potentially enhancing tumor growth and/or metastatic evolution. *HBEGF* and *AREG* genes are underlined.

Collectively, these results support that PR isoforms may differentially contribute to carcinogenesis-associated cellular functions mainly in a ligand-sensitive manner and highlight the major impact of imbalanced PRA/PRB ratio in such processes.

## Discussion

The presence of PR and more importantly the PRA/PRB ratio directly influence breast cancer phenotype, even in the absence of P4 [47]. Breast cancer patients with PRA dominant tumors have poorer disease-free survival rates [48]. If PR isoforms contribute towards pathogenesis, aggressive phenotype of PRA as compared to PRB-rich tumors might be due to major alterations in P4 signaling via PRA *vs* PRB isoform. The mechanisms by which PR contributes to such tumorigenic processes are not fully known in part due to the lack of appropriate cellular models. *In vitro* transcriptomic studies performed in separate cell lines conditionally expressing either PRA or PRB or both [24]–[26], [49]–[51] have highlighted distinct transcriptional properties of PR isoforms depending on the presence or absence of P4. Upon P4 exposure, PRB was reported to be a much stronger transcriptional factor than PRA alone in T47D-iYA and -iYB cell lines [25]. Conversely, in the absence of P4, PRA plays a prominent role since it regulates a much larger distinct gene population as compared to PRB alone [25]. The strategy of conditional expression of steroid hormone receptors including PR by the use of inducible systems has been reported [52]–[56]. However, none of these cellular models allows PRA and/or PRB expression in the same cells and thus could not permit studying the role of PRA/PRB ratio in cell signaling in the absence or presence of P4. To circumvent these drawbacks, it was necessary to develop a new strategy in the form of bi-inducible system. The relative contribution of PRA, PRB homo- and hetero-dimers in P4 signaling could be investigated with more confidence since such cells serve as their own controls when cultured in the absence of inducer(s). Moreover, mammary breast cancer MDA-MB-231 (ERα−, PR−) cells were selected hence enabling investigation of PR specific events independently of estrogen-dependent signaling. Of note, we cannot exclude that the absence of ERα (and estrogens) might modify expression of coregulators required for PR signaling and thus influence transcriptional responses to PR isoforms. However, in ER+PR+ cells, cancer-related modifications might also influence the cross talks of ER and PR signalings in an unpredictable manner depending on cancer phenotype and cell type. Taking into account these limitations, nevertheless our studies provide new insight for the understanding of the functional consequences of imbalanced PRA/PRB ratio in the context of metastatic mammary cancer cells. In the future, the iPRA and iPRB cell lines can also be used to conditionally express ERα or any co-regulator to investigate their impact on PR isoform specific functions.

To validate our strategy of bi-inducible PR isoforms expression, we focused on several aspects of PR signaling highlighting the critical role of PRA/PRB ratio in transcriptional regulation and cross-talk with growth factor stimuli. PR isoforms are considered as prognostic markers of breast cancer development and metastasis independently of the progestational status [48], [57]. P4 exerts antiproliferative effects in mammary PR+ cells [40] and few proliferating mammary epithelial cells (MEC) express PR [58], [59]. Recently, the role of P4 signaling in mammary carcinogenesis via paracrine mechanism gained strength since various independent studies have demonstrated that P4 drives proliferation of PR(−) MEC leading to carcinogenesis via receptor activator of NF-kB ligand (RANKL), a tumor necrosis factor family member, and its receptor (RANK) [40], [60], [61]. Consistent with previous studies [62], we found that both P4 and antiprogestin RU486 exerted antiproliferative effects when either or both PR isoforms were expressed. Interestingly RU486 but not P4 exhibited stronger antiproliferative effects in PRA− as compared to PRB-expressing cells suggesting that PRA/PRB expression ratio influences the antiproliferative efficacy of antiprogestins. This should be taken into consideration during therapeutic applications involving antiprogestins.

To further validate our cell line as a new model for breast cancer studies, we selected *HBEGF* and *AREG* genes known to play important role in breast tumorigenesis [63]–[66] and proposed as therapeutic targets for various human cancers including breast cancer [67]. PR-dependent transcriptional regulation of *HBEGF* gene has been reported [68], [69]. Previous studies using Ishikawa cells stably expressing either of the PR isoforms have identified *AREG* to be a PRB- but not PRA-regulated gene [44]. We show that *AREG* expression is regulated not only by liganded-PRB but interestingly also by unliganded PRA. PR transcriptional activity is known to be highly influenced by EGF stimulation [46], [70]. The differential roles of PR isoforms in regulating *HBEGF* and *AREG* expression under P4 and EGF stimulation have not been described. Our results on *AREG* transcription revealed that EGF strongly potentiates P4-induced *AREG* expression uniquely via PRB similar to synergetic effect of P4 and EGF on PRB-mediated transactivation from exogenous promoters. Interestingly co-expression of PRA compromised such potentiating effect of EGF. In contrast, P4 and EGF exerted opposing effects on *HBEGF* transcription, P4 decreased while EGF increased the *HBEGF* expression in PRA or PRB expressing cells. The effect of P4 on this gene was in contrast to previous observations performed in T47D cells [68], showing that P4 enhanced HBEGF transcript via PRB but not PRA. This effect has been connected to Cdk2-dependent phosphorylation at Ser81 of PRB that is absent in PRA. Such discordance highlights that difference in kinase activities frequently observed in cancer cells might strongly influence PR signaling. Although P4 exerted antiproliferative effect in PR expressing cells, a noteworthy finding of our study is that unliganded PRA increased *AREG* expression that may provide continuous paracrine growth stimuli to adjacent PR(−) cells. Based on our results, it is tempting to speculate that PRA controls cell proliferation by two mechanisms, a cell-intrinsic mechanism i.e. by decreasing cyclin D1 expression and by paracrine mechanism by increasing *AREG* expression. Moreover, we highlight that elevated PRA/PRB ratio is associated with increased *AREG* expression that could be inhibited by antiprogestin RU486. Given the involvement of *AREG* in cancer development/metastasis as a paracrine growth stimulus, aggressive phenotype of PRA rich tumors might at least in part be attributed to elevated *AREG* levels which may enhance the proliferation of nearby PR negative cells.

To investigate the differential role of PRA and PRB on gene transcription and evaluate the impact of unbalanced PRA/PRB ratio on breast cancer, it was necessary to perform genome-wide transcriptomic studies. The impact of conditional expression of PRA and/or PRB was examined after 6 h treatment by ligand or not and in the absence of protein synthesis inhibitor. Such condition was expected to limit as much as possible the effects of trans-acting protein neosyntheses or variation in cell cycle-dependent factors impacting transcription as well as any functional impact of differential turnover of PR isoforms [23]. Our data are thus relevant for the early differential actions of PR isoforms but not for their delayed functions. Moreover, our aim was to discriminate in the same cells the qualitative transcriptional changes induced by highly imbalanced as compared to normal PRA/PRB ratio. This allowed us to predict that lack of one isoform frequently observed in breast cancers may paradoxically result in selection of new transcriptional targets as well as in suppression of regulations depending on PRA and PRB co-expression. It is likely that exploring intermediary PRA/PRB ratios found in most cancer cells would not allow to identify new regulated genes as compared to the lists included herein. PRA/PRB ratio can be disrupted in the range 0–100 in PR+ human breast tumors, mainly due to exacerbated down-regulation of one isoform rather than imbalanced overexpression [71]. Transcriptional outcomes of such variable ratio could be evaluated in the future by extensive experiments using iPRAB cells, and more interestingly in xenographted mice.

Our results show that PRA and PRB target directly or indirectly three distinct classes of genes that we defined as either ligand-independent (Class I) or ligand-dependent (Class II) or responding to mixed conditions (Class III) in iPRAB cell line. This could be compared to recent microarray studies in two osteosarcoma cell lines (U2OS) conditionally expressing ERα or ERβ [72], showing that ERα regulates only one class of genes (liganded ER) while ERβ targets 3 distinct classes in a manner similar to PRA and PRB. Such transcriptional selectivity of ERβ was essentially determined by promoter context rather than variation in DNA binding. The large population of PR-regulated genes insensitive to P4 shows that PR can be activated by stimuli other than hormone. Such activity might be related to phosphorylation-dependent activation of PR induced by EGF treatment [46]. This is highly suggestive for an important role of ligand-independent PR post-translational modifications while ligand-induced phosphorylation, sumoylation and acetylation have been shown to strongly influence PR transcriptional properties by modifying PR interaction with other transcriptional factors as well as transcriptional coregulators [20], [21], [73]–[79]. Other interesting findings concern the genes regulated only when both PR isoforms are co-expressed. Ligand-induced PR homodimers and heterodimers might exert distinct trans-acting effects on target gene selection likely dependent on promoter context. Furthermore, since dimers cannot be stably assembled in ligand-free condition, the cooperativity of PRA and PRB monomers may be also required to trigger promoter activity of certain genes through mechanisms that are not yet defined. The third group of genes that we defined as mixed responsive genes, differentially responded to ligand-free or hormone-bound condition, since P4 either potentiated or reduced transcriptional response to unliganded PR. In addition, large subsets of genes were inversely regulated by unliganded and liganded isoforms showing that transcriptional mechanisms involving PR are extremely diversified and complex. It is interesting to note that PRB as well as PRA are able to impair activity of the other isoform in both conditions of ligands for 30% of the genes sensitive to both isoforms. However, the majority of genes commonly targeted by PRA and PRB are regulated in concert excluding any transrepressive mechanism. Our results thus suggest the high degree of PR isoform selectivity and hormonal condition found in PR responsive genes as an intricate function of PRA and PRB relative expressions and promoter structure. Further studies are now needed to discover the determinants of the underlying mechanisms.

Importantly, our functional analysis of PR-regulated gene population identifies signaling pathways and cellular functions that are differentially impacted by PR isoforms supporting the notion that deregulated isoform expression may contribute to cancer progression. Expression of cancer associated genes is predominantly regulated by PRA as compared to PRB consistent with aggressive phenotype of PRA-dominant breast tumors [48]. The gene lists and classification given as Supplementary data might be the basis for future studies and allow formulating some interesting hypotheses. For example, among these genes, we found that *TNFRSF11B,* coding a decoy receptor of RANKL that neutralizes this ligand, was down-regulated by both liganded PR isoforms, while interestingly *TNFRSF11A*, coding the functional receptor for RANKL, was specifically upregulated by liganded PRA. Such dual regulations suggest the possibility of P4-dependent amplification of cellular response to RANKL, contributing towards PR-sensitive carcinogenesis as previously reported [60]. PRA and PRB expressions, independently of estrogen-dependent signaling, impact 200 distinct genes known to be involved in breast cancer and/or metastasis, from which 90 are regulated in unfavorable direction, with a predominant role of PRA. This is due in particular to the larger population of genes regulated by PRA targeting cell proliferation processes as compared to PRB when expressed alone. Importantly, the exclusive expression of PRA or PRB increased the impact of hormone on PR-dependent pathways playing a major role in cancer. Refining the analysis of 185 key genes moved by a lack of either PRA or PRB expression as compared to PRAB cells, we determined the transcriptional changes of respectively 54 and 25 key genes potentially favoring tumor proliferation and/or metastasis. Therefore, our studies predict a poor outcome of PRA+/PRB− as well as PRA−/PRB+ breast cancers contrasting with the consensus model. Functional studies are needed to confirm these reciprocal drawbacks *in vivo*.

In conclusion, our newly established bi-inducible breast cancer cellular model is of broad interest since it allows mechanistic understanding of how target cells behave with PR ligand and with other PR activators such as EGF under normal and pathological contexts. Importantly the role of varied PRA/PRB expression ratio in breast cancer development and metastasis can be explored in mice xenografted with iPRAB cells and exposed to varying amounts of Dox and/or RSL1 to control PRA/PRB ratio. This might be of particular pharmacological interest for screening and characterization of new PR antagonists for their PR isoform selective inhibitory properties. We believe that our cell line associated with transcriptomic data will help future investigations in defining the role of identified genes in pathological conditions including breast cancer.

## Materials and Methods

### Plasmid Constructions

The bi-inducible expression system used for differential expression of PR isoforms was a combination of the original Rheoswitch [80] and the T-Rex (Invitrogen) systems. For the former system, the original plasmids pZX-LR and pZX-luc were kindly provided by Dr. Claude Labrie [53]. For the T-Rex system, the original plasmids were obtained from Invitrogen. Both Rheoswitch and T-Rex were combined in a new single plasmid pZX-TR as schematized in Supplementary Fig. S1, constitutively expressing Rheoactivator, Rheoreceptor, Tet-R repressor and the Zeocin resistance genes. Human PRA and PRB cDNAs were obtained from pSG5-hPRA and pSG5-hPRB [81]. Vectors conditionally expressing hPRA (pGALp-hPRA) under the control of Rheoswitch Ligand 1 (RSL1), and hPRB (pTO-hPRB) under the control of doxycyclin (Dox) were constructed (schematized in Supplementary Fig. S1). The pGAL-luc reporter gene vector used for screening steps expressing luciferase under the control of 6xGAL4uas elements was constructed from pGL3 (Promega) and pZX-LR. For similar uses, the pTO-luc reporter gene vector was constructed from pGL3 and pcDNA4-TO (Invitrogen). PR-specific reporter gene plasmid (PRE_2_-Luc) expressing luciferase under the control of two progesterone responsive elements (PRE_2_) was used for screening steps and transcription analyses as previously described [81]. Complete plasmid sequences and detailed multistep constructions of all plasmids are available upon request.

### Production of Inducible Cell Lines

Approximately, 2×10^6^ MDA-MB-231 cells were transfected with 2 µg of pZX-TR plasmid using LipofectAMINE 2000 (Invitrogen). For functional analysis of inducible systems, approximately 150 zeocin resistant clones were transfected with either pGal4p-luc or pTO-luc vectors and incubated with or without RSL1 (0.5 µM) or Dox (2 µM) during 24 h. Ten dually positive clones having highest inducible expression and minimum background for both systems were selected and tested by western blot for the expression of the three regulator proteins. Clone 250 was selected to generate secondary stable cells following co-transfection by either pGal4p-PRA/neomycin (iPRA) or pTO-PRB/blasticidin (iPRB) or both plasmids (iPRAB). After 48 h, cells were plated in medium containing respective selection antibiotic(s) and 45 clones resistant to selection antibiotics were amplified and screened for PR isoforms expression following 24 h incubation without or with RSL1 and/or Dox by immunobloting. Ten clones with minimum background and maximum PR isoform expression following inducer(s) exposure were further analyzed by immunocytochemistry for clonal expansion after 3 consecutive passages. All these clones gave consistent inducible expression of each isoform and clone iPRAB-38 conditionally expressing comparable levels of PRA and PRB to homogeneity after 10 passages was selected for this study.

### Cell Culture and Reagents

Human breast cancer cells MDA-MB-231 (ER−, PR−) were obtained from the American Type Culture Collection (ATCC, HTB-26) and were routinely cultured in Dulbecco’s Modified Eagle’s Medium (DMEM) as described previously [82]. Stable cell lines were grown in the presence of zeocin (100 µg/ml, Invitrogen) for selection of pZX-TR, neomycin (200 µg/ml, Sigma) for pcDNA3-Galp-hPRA (iPRA), blasticidin (2 µg/ml, Euromedex) for pcDNA6-TO-hPRB (iPRB). The non-steroidal ligands RSL1 (New England BioLabs) and/or doxycycline (Dox) (Sigma) were added at the indicated concentration for induction of PRA and/or PRB. For hormonal treatments, ligands or vehicle (ethanol) were added in steroid-free medium [82] at the indicated concentration, including progesterone, R5020 (17,21-dimethyl-19-norpregna-4,9-dien-3,20-dione), or RU486 (11β-(4-Dimethyl-amino)-phenyl-17β-hydroxy-17-(1-propynyl)-estra-4,9-dien-3-one). Progesterone, R5020, RU486 and EGF were purchased from Sigma.

### Luciferase Activity Assays

PRA and/or PRB expression was induced using RSL1 (0.5 µM) and/or Dox (2 µM) during 24 h in steroid free medium. Cells were transfected with indicated reporter gene (100 ng) and β-galactosidase (10 ng) plasmids as previously described [23]. After 24 h, cells were incubated with indicated ligands and cells were collected with the Passive Lysis Buffer (Promega). Luciferase activity was measured with luminometer Victor (Perkin Elmer) and compared to total protein content in cell lysate quantified by the bicinchoninic acid (BCA) assay (Interchim) to control homogeneity of transfections. The results were normalized to β-galactosidase activity or total protein concentration as indicated. The data are presented as means ± SE of six independent cell cultures (n = 6).

### Immunoblotting

Western blot analysis was performed as previously described [82]. Antibodies used were, anti-PRA and anti-PRB (NCL-PGR-312, Novocastra Laboratories), anti-α-tubulin antibody (Sigma), horseradish peroxidase-conjugated goat anti-mouse or anti-rabbit secondary antibody (Vector Laboratories, Burlingame, CA). Target proteins were detected using ECL Plus reagent (GE Healthcare) and visualized by chemiluminescence.

### PR Quantification by Ligand Binding Assays

The iPRAB cells were incubated with RSL1 and/or Dox or vehicle during 24 h in steroid-free medium. Parental MDA-MB-231 (PR−) cells were used as control. Cytosols were prepared and incubated at 4°C with 10 nM^ 3^H-R5020 in the absence or presence of unlabeled R5020 (1 µM) for 2 h. Bound and unbound steroids were separated by the dextran-charcoal technique and radioactivity was counted in supernatant. The results from triplicate experiments were expressed as fmol R5020 binding sites per mg of total proteins measured by BCA assays.

### Immunocytochemical Assays

Cells were fixed with 4% paraformaldehyde and permeabilized 30 min with PBS containing 0.5% Triton X100. Cells were incubated with primary anti-PR antibody (Novocastra) overnight at 4°C and for 30 min with an Alexa 488-coupled anti-mouse IgG secondary antibody. After analysing the cells with fluorescent microscopy, pictures acquisition was performed at 20× magnitude for 160 ms with imaging Qcapture Pro version 5.1 (Q Imaging Inc.).

### Quantitative Real-time RT-PCR

Total RNAs extraction and real-time qRT-PCR procedures have been previously described [81]. Primers sequences are presented in Supplementary Table S1. All samples were analyzed in duplicate from at least three independent cell cultures. The relative expression level of each gene transcript was measured as compared to the corresponding cloned amplicon and normalized with 18S RNA level.

### Proliferation Assays

Approximately 5000 iPRAB-38 cells per well were plated in 96-well plates for 24 h. On Day 0, medium was replaced with 5% DCC medium with inducer RSL1 (0.5 µM) and/or Dox (2 µM) for PRA and/or PRB expression along with indicated steroid(s). Same treatment was repeated on Day 2 and 4. Cell proliferation was determined on Day 1, 3 and 5, by adding 20 µl of Cell Titer96 AqueousOne solution (Promega) into each well containing 100 µl of culture medium. The plates were incubated at 37°C for 1 h and absorbance value at 490 nm (A490) was measured using photometer (Victor 378, Perkin Elmer).

### Gene Expression Profiling

The iPRAB cells were grown either in the absence (O) or presence of 0.25 µM RSL1 (A) or 1 µM Dox (B) or both (AB) inducers for 24 h and then treated by either vehicle (−) or 10 nM progesterone (+) for 6 h, leading to prepare the O−, O+, A−, A+, B−, B+, AB−, AB+ sample extracts, corresponding to expression of no PR, PRA, PRB, PRA + PRB respectively bound (+) or not (−) with P4. RNA extraction was done using Trizol protocol and purified with Qiagen column (Rneasy micro). The quantity and purity of the extracted RNA was evaluated using a NanoDrop spectrophotometer and its integrity measured using Lab-on-a-chip Bioanalyser 2000 technology (Agilent Technologies, Palo Alto, CA, USA), based on the 28S/18S ribosomal RNAs ratio. Labeling of RNA samples was done according to Agilent oligo Cy5 or Cy3 probes labeling protocol using the Agilent Low Input QuickAmp labelling kit for dye swap strategy. Hybridization of each Cy5/Cy3 cRNA mixture was carried out to the Agilent Human Whole Genome Oligo Microarray format 4×44 K (G4112F A AGIL_28, Agilent Technologies) for 17 hours at 65°C. Following scanning with Agilent G2505C DNA Microarray scanner using default parameters (20 bits mode, 3 µm resolution, at 20°C in low ozone concentration environment), the data were extracted using Feature Extraction software (v 10.5.1.1 Agilent) with default setting, and normalized using protocol GE2 105. Data from all hybridizations were analyzed with Rosetta Resolver software (version 7.2.2.0) and statistical analysis for quality test was performed to control homogeneity of hybridization signals. Dye swap arrays were combined using an error-weighted average in order to avoid dye incorporation bias. A list of differentially expressed transcripts was extracted from each hybridization for absolute fold change (FC) >1.5 and p-value <10^−5^. The 16 lists were joined, and ten probes significantly positive with RNA sample obtained from hormone-treated cells in the absence of PR inducers (O+/O−) were eliminated from the list. Redundant positive probes corresponding to the same gene were grouped to control homogeneity, and only one representative response was conserved allowing counting the real number of genes. The final list included 953 protein-coding and 46 non-protein coding RNA corresponding to miRNAs and miscellaneous approved RNA. Raw microarray data have been deposited in compliance to MIAME guidelines at ArrayExpress database (http://www.ebi.ac.uk/arrayexpress), with accession number E-TABM-1215 release date June 11, 2012.

### Classification of PR-regulated Genes

Gene subsets corresponding to each combination of responses analyzed by microarrays were defined from Venn diagrams indicating the number of the included genes. For this, an absolute FC <1.3 or a p-value >10^−3^ found in the combined dye swap for a given gene was considered as a null response to the corresponding experimental condition (FC arbitrary set to 1). For each conditional subset, a hierarchical clustering was additionally performed through complete linkage mode from log_2_ (ratio) values using Genesis software (http://genome.tugraz.at) to generate the final heat maps designing the up- and down-regulated gene clusters. Such sequential ordering was necessary for the heat map classification instead of classical hierarchical clustering since the reference samples were different for each hybridization experiment. In addition, we multiplied the expression ratios experimentally obtained at a p-value <10^−3^ for ligand-specific and ligand-free conditions (i.e. A+/A− × A−/O−, B+/B− × B−/O−, AB+/AB− × AB−/O−) to directly compare all PR-expressing samples to the same O− reference (i.e. A+/O−, B+/O−, AB+/O−, A−/O−, B−/O−, AB−/O−). We further calculated the ratio A−/AB−, B−/AB−, A+/AB+ and B+/AB+ to determine the relative impact of a defect in one isoform expression as compared to the equimolar expression of PRA and PRB.

### Functional Analyses of Microarray Data

Ingenuity systems Pathways Analysis (IPA) (Ingenuity® Systems, http://www.ingenuity.com) was used for functional mapping and canonical pathways through the IPA knowledge database. IPA p-value were calculated for each group of genes through right-tailed Fisher’s exact test, and indicate the probability that each biological function and/or disease assigned to the data set is due to chance alone. The cut-off for functional analysis were transcriptional FC >1.3 and IPA p-value <0.05. Protein ANalysis THrough Evolutionary Relationships (PANTHER) classification (http://www.pantherdb.org) was used to determine the protein families, biological processes and pathways according to their respective representation in the whole human genome by a p-value assessed by the binomial statistic method. A gene category was considered as significantly over represented in a data set for a p-value <0.05 and a fractional difference ([n-N]/N) between the number of genes mapped to this category in the list (n) and the number expected by random chance (N) higher than 1.3. Such preselected lists were then compared to the whole transcriptional pattern regulated by PR irrespective to isoform and ligand condition, allowing to define the relative impact of isoform conditional expression and ligand status on PR-dependent pathways and cellular functions (% of total PR-regulated genes in the category).

### Statistical Analysis

Data are expressed as mean ± SEM. Non parametric Mann-Whitney test was used to determine significant differences between groups using the computer software Prism 4 (GraphPad Software, San Diego, CA). Statistical significance is indicated by 1–3 symbols (stars or crosses) corresponding to *P*≤0.05 or ≤0.01 or ≤0.001 respectively.

## Supporting Information

Figure S1 **The strategy employed for conditional PR isoforms expression from bi-inducible promoter.** The coding sequences of the regulatory proteins required for RheoSwitch (Rheoreceptor, Rheoactivator) and T-Rex (Tet Repressor) systems were inserted in the same plasmid named pZX-TR, and primary stable cell lines were established as described in *Materials and Methods*. For secondary stable cell lines, PRA or PRB expression was placed under the control of the indicated promoters sensitive to RheoSwitch or T-Rex modulators respectively. In uninduced state, PRA or PRB expression is silenced, while the addition of RSL1 or Dox selectively induces the transcription of PRA or PRB. Three cell lines were established, iPRA, iPRB, and iPRAB expressing PRA or PRB or PRA+PRB respectively.
(PDF)

Figure S2 **The character of iPRAB cells for conditionally expressing PR isoforms is conserved following 22 passages.** The iPRAB cells were cultured up to 22 passages and immunoblot analysis was performed following 24 h of exposure to vehicle or indicated inducer(s) as described in *Materials and Methods*.
(PDF)

Figure S3 **PR isoforms undergo ligand-dependent modifications in iPRAB cells.** The iPRAB cells were incubated with indicated inducers during 24 h, then treated with P4 (10 nM) or RU486 (10 nM) for another 24 h, and were analyzed by western blot. (A) PRA and PRB were detected using anti-PR antibody (Novocastra). P4 or RU486 respectively induced down-regulation or stabilization of PRA and PRB as compared to vehicle condition. (B) P4 induced electrophoretic upshifts of polyphosphorylated PRA and PRB detected as in A. (C) PRA and PRB were detected using an antibody (Affinity BioReagent) directed against PR phosphorylated species (pS130-PRA, pS294-PRB), showing that key Ser294/130 is phosphorylated. PRA and PRB profiles are presented along with tubulin sample loading control.
(PDF)

Figure S4 **RSL1 does not induce AREG expression in parental MDA-MB-231 or clone 250 cells.** Parental MDA-MB-231 (PR−) or clone 250 cells (pZX−TR+, PR−) were cultured in the presence of vehicle or RSL1 (0.5 µM) during 24 h and qRT-PCR analysis was performed for AREG transcript levels as described in *Materials and Methods*.
(PDF)

Figure S5 **PRA enhances AREG expression in stably transfected MDA-MB-231 or Ishikawa cells.** (**A**) MDA-MB-231 or Ishikawa cells stably expressing PRA or not were cultured under similar conditions and qRT-PCR analysis was performed for AREG transcript levels as described in *Materials and Methods*. The data (mean ± SEM) from three independent cell cultures measured in duplicate is presented as fold change in AREG transcript levels in PRA expressing cells as compared to PR− cells. (**B**) MDA-iPRAB cells were treated by RSL1 or vehicle for 24 h in 6-well plates, and then the medium was replaced by fresh DCC without ligand. Following incubation for 24 h, AREG protein was quantified in cell lysates by ELISA (DY262, R&D systems), and results were expressed as pg/ml of cell lysate (mean ± SEM from 6 experiments, unpaired t test). Total protein concentration was 2 mg/ml.
(PDF)

Figure S6 **Differential impact of PRA and PRB expression on molecular pathways.** Functional analysis of microarray data was performed using the Ingenuity system database (IPA) for each conditional PR isoform expression. IPA p-values were calculated using right-tailed Fisher’s exact test (cut-off : transcriptional FC ±1.3 and p-value <0.001; IPA p-value <0.05). For each pathway and condition, an impact factor (IF) on whole cell transcriptome was calculated as –log(IPA p-value) and represented according to the indicated blue color scaling. Ratio (%) corresponding to the number of PR isoform-dependent genes targeting a given pathway as compared to the total number of known human genes involved in this pathway is represented according to the indicated green color scaling. The indicated IPA pathways impacted by progesterone-independent (vehicle) and/or progesterone-dependent genes (progesterone) are listed according to each conditional PR isoform expression in decreasing order of IF.
(PDF)

Figure S7 **Relative impact of PRA ant PRB on PR-regulated biofunctions.** Functional analysis of microarray data was performed using PANTHER system from each conditional expression gene list (A, B, AB) obtained for unliganded (−P4) or liganded (+P4) PR isoforms as described in *Material and Methods*. Following comparison with the whole PR-regulated genes, p-values were obtained using a binomial test, and the most significant functions (p-value <0.01) were extracted. Fraction of genes (%) for each condition participating to a given PR-regulated function targeted by at least 10 genes is mapped on a polar chart (100% relates to all PR-regulated genes impacting the function irrespective to ligand and isoform expression level). (A) Biological pathways, (B) Cellular processes.
(PDF)

Table S1 **Primer sequences for qPCR experiments.**
(PDF)

Table S2 **Comparison with previous microarray data.** List of 172 genes regulated in iPRAB cell line that have been at least once reported as PR targets.
(XLS)

Table S3 **Results of microarray analyses.** List of 999 PR isoform-dependent differentially regulated genes along with the transcriptional fold changes (FC) and p-values obtained by combined dye swap microarray analysis of iPRAB cell line.
(XLS)

Table S4 **Functional annotations of PR-responsive genes.** Complete gene ontology annotations of PR isoform-regulated genes giving the opportunity to compare transcriptional fold changes and key biological functions. The classification was processed using IPA and PANTHER analysis systems as described in *Material and Methods*.
(XLS)

Table S5 **Functional analyses of microarray data.** The MS Excel workbook includes statistical results for the impacted biological processes (categories), functions and canonical pathways and the corresponding lists of PR isoform-regulated genes obtained through IPA knowledge database as compared to the whole human genome. In addition, PANTHER system was used to analyze the differential contribution of PR isoforms in the functions regulated by the whole PR target genes population.
(XLS)

Table S6 **List of PR-regulated genes impacting cancer.** Transcriptional changes induced by expression of PRA and/or PRB in up or down direction that might potentially either decrease (anti) or enhance (pro) tumor growth and/or metastatic evolution. Cancer-related genes were primary selected using IPA system with a cut-off FC ±1.3 and a p-value <10^−3^. Key genes involved in ‘molecular mechanisms of cancer’ category are indicated (*). The *HBEGF* and *AREG* genes studied in the article are underlined.
(PDF)
