# Supplementary material for: Differential Regulation of Breast Cancer-Associated Genes by Progesterone Receptor Isoforms PRA and PRB in a New Bi-Inducible Breast Cancer Cell Line
Source: PLoS One. 2012 Sep 24;7(9):e45993. doi: 10.1371/journal.pone.0045993 (PMC3454371; doi:10.1371/journal.pone.0045993)

Figure S1

The strategy employed for conditional PR isoforms expression from bi-inducible promoter. The coding sequences of the regulatory proteins required for RheoSwitch (Rheoreceptor, Rheoactivator) and T-Rex (Tet Repressor) systems were inserted in the same plasmid named pZX-TR, and primary stable cell lines were established as described in *Materials and Methods*. For secondary stable cell lines, PRA or PRB expression was placed under the control of the indicated promoters sensitive to RheoSwitch or T-Rex modulators respectively. In uninduced state, PRA or PRB expression is silenced, while the addition of RSL1 or Dox selectively induces the transcription of PRA or PRB. Three cell lines were established, iPRA, iPRB, and iPRAB expressing PRA or PRB or PRA+PRB respectively.

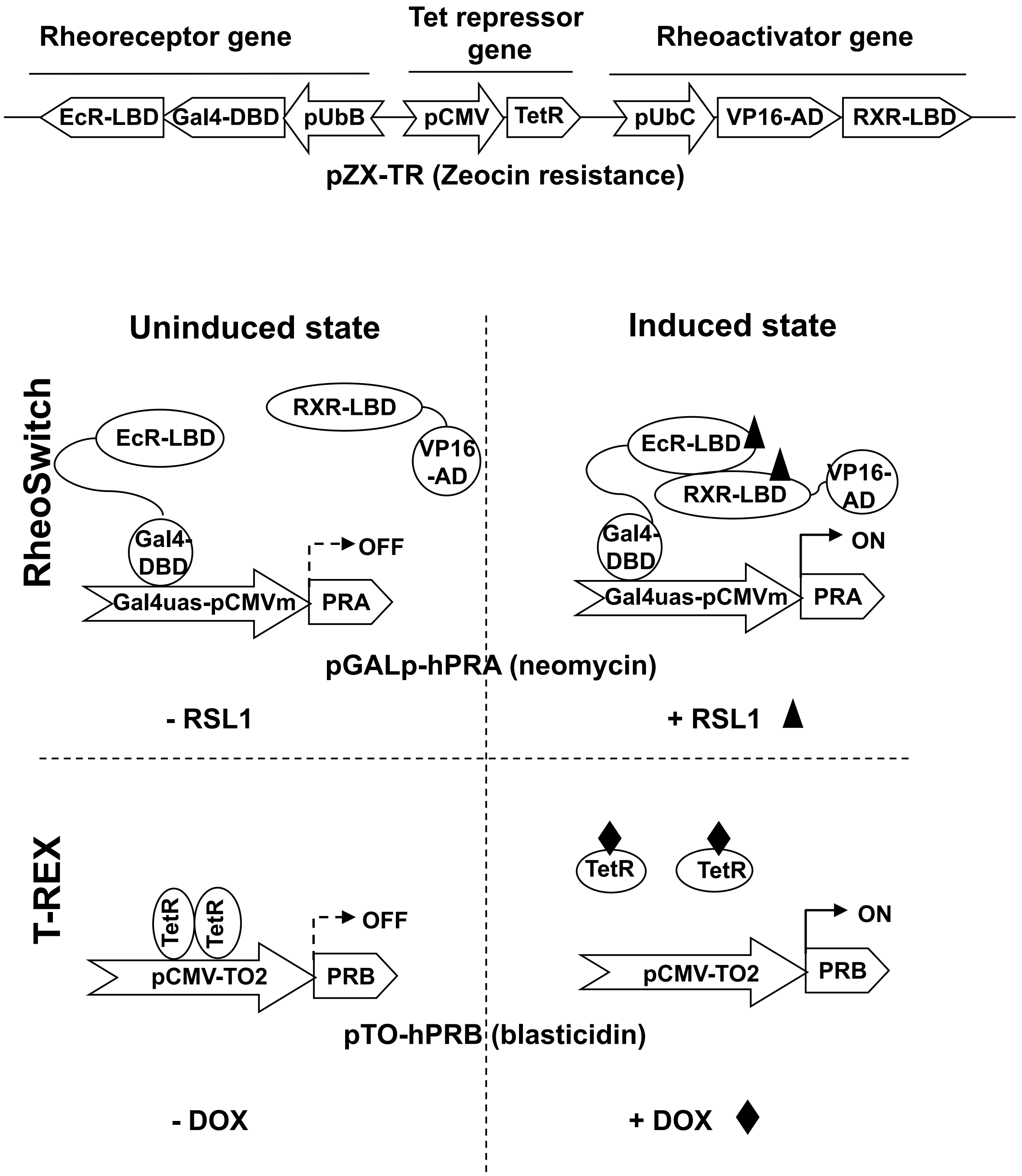

Supplement: Figure S1 — The strategy employed for conditional PR isoforms expression from bi-inducible promoter. The coding sequences of the regulatory proteins required for RheoSwitch (Rheoreceptor, Rheoactivator) and T-Rex (Tet Repressor) systems were inserted in the same plasmid named pZX-TR, and primary stable cell lines were established as described in Materials and Methods. For secondary stable cell lines, PRA or PRB expression was placed under the control of the indicated promoters sensitive to RheoSwitch or T-Rex modulators respectively. In uninduced state, PRA or PRB expression is silenced, while the addition of RSL1 or Dox selectively induces the transcription of PRA or PRB. Three cell lines were established, iPRA, iPRB, and iPRAB expressing PRA or PRB or PRA+PRB respectively. (PDF) [file pone.0045993.s001.pdf]
