# Supplementary material for: Differential Regulation of Breast Cancer-Associated Genes by Progesterone Receptor Isoforms PRA and PRB in a New Bi-Inducible Breast Cancer Cell Line
Source: PLoS One. 2012 Sep 24;7(9):e45993. doi: 10.1371/journal.pone.0045993 (PMC3454371; doi:10.1371/journal.pone.0045993)

Figure S2

The character of iPRAB cells for conditionally expressing PR isoforms is conserved following 22 passages. The iPRAB cells were cultured up to 22 passages and immunoblot analysis was performed following 24 h of exposure to vehicle or indicated inducer(s) as described in *Materials and Methods*.

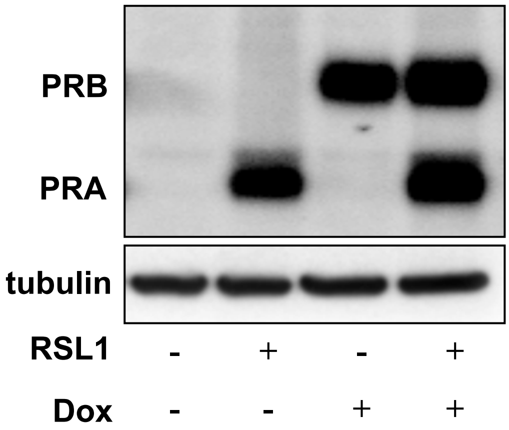

Supplement: Figure S2 — The character of iPRAB cells for conditionally expressing PR isoforms is conserved following 22 passages. The iPRAB cells were cultured up to 22 passages and immunoblot analysis was performed following 24 h of exposure to vehicle or indicated inducer(s) as described in Materials and Methods. (PDF) [file pone.0045993.s002.pdf]
