# Supplementary material for: Differential Regulation of Breast Cancer-Associated Genes by Progesterone Receptor Isoforms PRA and PRB in a New Bi-Inducible Breast Cancer Cell Line
Source: PLoS One. 2012 Sep 24;7(9):e45993. doi: 10.1371/journal.pone.0045993 (PMC3454371; doi:10.1371/journal.pone.0045993)

Figure S3

PR isoforms undergo ligand-dependent modifications in iPRAB cells. The iPRAB cells were incubated with indicated inducers during 24 h, then treated with P4 (10 nM) or RU486 (10 nM) for another 24 h, and were analyzed by western blot. (A) PRA and PRB were detected using anti-PR antibody (Novocastra). P4 or RU486 respectively induced down-regulation or stabilization of PRA and PRB as compared to vehicle condition. (B) P4 induced electrophoretic upshifts of polyphosphorylated PRA and PRB detected as in A. (C) PRA and PRB were detected using an antibody (Affinity BioReagent) directed against PR phosphorylated species (pS130-PRA, pS294-PRB), showing that key Ser294/130 is phosphorylated. PRA and PRB profiles are presented along with tubulin sample loading control.

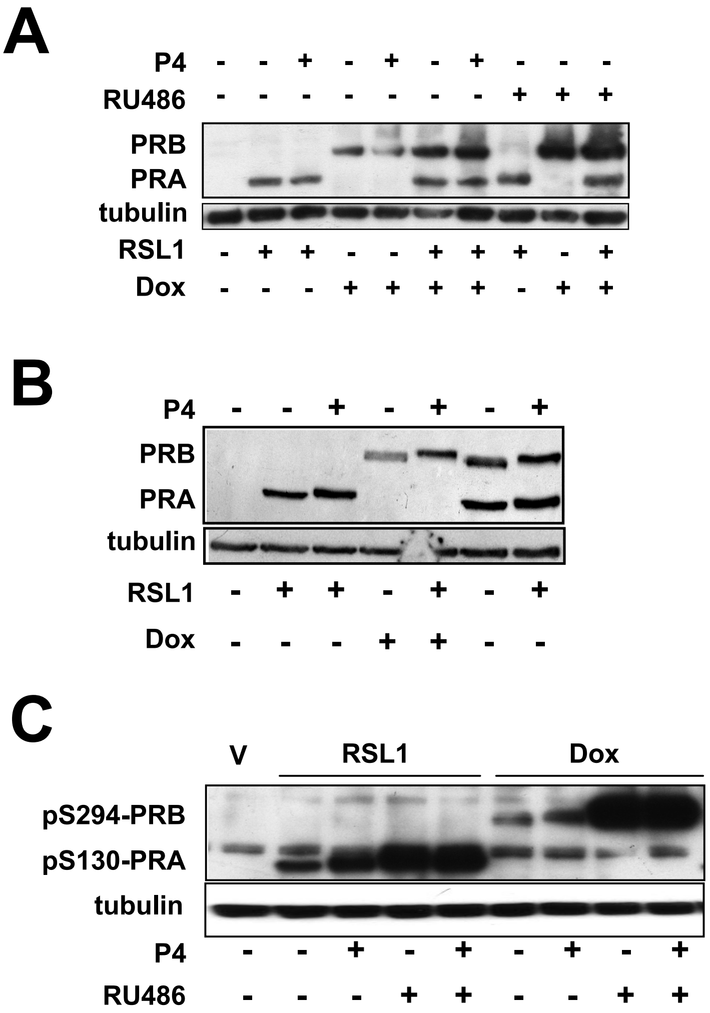

Supplement: Figure S3 — PR isoforms undergo ligand-dependent modifications in iPRAB cells. The iPRAB cells were incubated with indicated inducers during 24 h, then treated with P4 (10 nM) or RU486 (10 nM) for another 24 h, and were analyzed by western blot. (A) PRA and PRB were detected using anti-PR antibody (Novocastra). P4 or RU486 respectively induced down-regulation or stabilization of PRA and PRB as compared to vehicle condition. (B) P4 induced electrophoretic upshifts of polyphosphorylated PRA and PRB detected as in A. (C) PRA and PRB were detected using an antibody (Affinity BioReagent) directed against PR phosphorylated species (pS130-PRA, pS294-PRB), showing that key Ser294/130 is phosphorylated. PRA and PRB profiles are presented along with tubulin sample loading control. (PDF) [file pone.0045993.s003.pdf]
