# Supplementary material for: Differential Regulation of Breast Cancer-Associated Genes by Progesterone Receptor Isoforms PRA and PRB in a New Bi-Inducible Breast Cancer Cell Line
Source: PLoS One. 2012 Sep 24;7(9):e45993. doi: 10.1371/journal.pone.0045993 (PMC3454371; doi:10.1371/journal.pone.0045993)

Figure S4

RSL1 does not induce AREG expression in parental MDA-MB-231 or clone 250 cells. Parental MDA-MB-231 (PR-) or clone 250 (pZX-TR+, PR-) cells were cultured in the presence of vehicle or RSL1 (0.5 μM) during 24 h and qRT-PCR analysis was performed for AREG transcript levels as described in *Materials and Methods*.

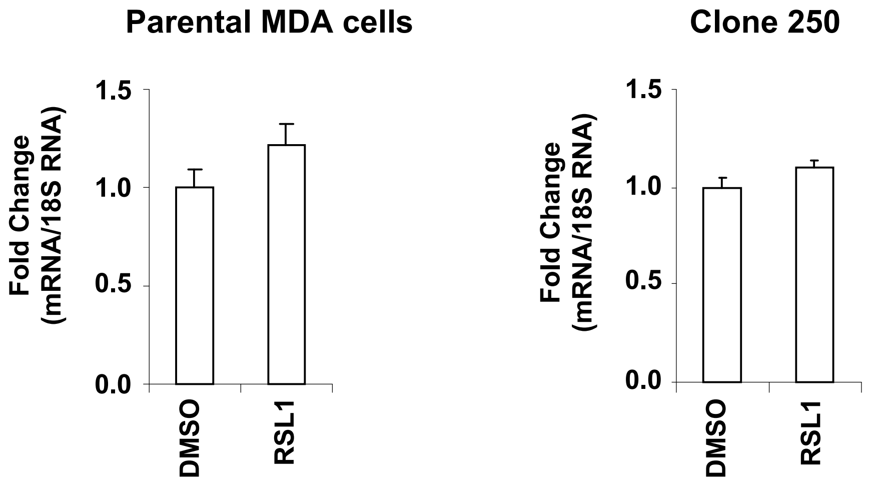

Supplement: Figure S4 — RSL1 does not induce AREG expression in parental MDA-MB-231 or clone 250 cells. Parental MDA-MB-231 (PR−) or clone 250 cells (pZX−TR+, PR−) were cultured in the presence of vehicle or RSL1 (0.5 µM) during 24 h and qRT-PCR analysis was performed for AREG transcript levels as described in Materials and Methods. (PDF) [file pone.0045993.s004.pdf]
