# Supplementary material for: Differential Regulation of Breast Cancer-Associated Genes by Progesterone Receptor Isoforms PRA and PRB in a New Bi-Inducible Breast Cancer Cell Line
Source: PLoS One. 2012 Sep 24;7(9):e45993. doi: 10.1371/journal.pone.0045993 (PMC3454371; doi:10.1371/journal.pone.0045993)

Figure S5

PRA enhances AREG expression in stably transfected MDA-MB-231 or Ishikawa cells. (**A**) MDA-MB-231 or Ishikawa cells stably expressing PRA or not were cultured under similar conditions and qRT-PCR analysis was performed for AREG transcript levels as described in *Materials and Methods*. The data (mean ± SEM) from three independent cell cultures measured in duplicate is presented as fold change in AREG transcript levels in PRA expressing cells as compared to PR- cells. (**B**) MDA-iPRAB cells were treated by RSL1 or vehicle for 24 h in 6-well plates, and then the medium was replaced by fresh DCC without ligand. Following incubation for 24 h, AREG protein was quantified in cell lysates by ELISA (DY262, R&D systems), and results were expressed as pg/ml of cell lysate (mean ± SEM from 6 experiments, unpaired t test). Total protein concentration was 2 mg/ml in all samples.

A

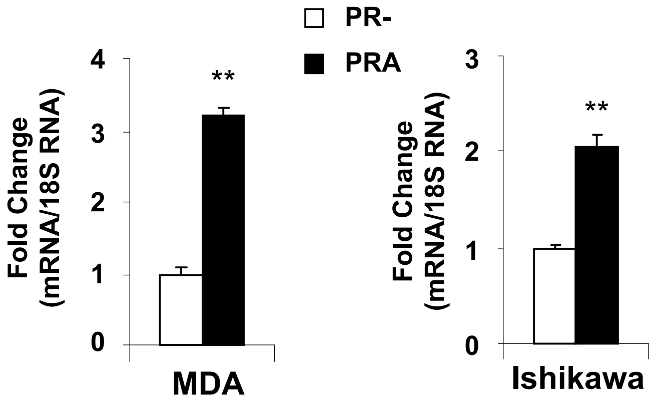

B

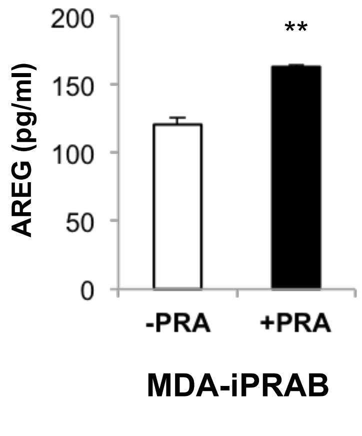

Supplement: Figure S5 — PRA enhances AREG expression in stably transfected MDA-MB-231 or Ishikawa cells. (A) MDA-MB-231 or Ishikawa cells stably expressing PRA or not were cultured under similar conditions and qRT-PCR analysis was performed for AREG transcript levels as described in Materials and Methods. The data (mean ± SEM) from three independent cell cultures measured in duplicate is presented as fold change in AREG transcript levels in PRA expressing cells as compared to PR− cells. (B) MDA-iPRAB cells were treated by RSL1 or vehicle for 24 h in 6-well plates, and then the medium was replaced by fresh DCC without ligand. Following incubation for 24 h, AREG protein was quantified in cell lysates by ELISA (DY262, R&D systems), and results were expressed as pg/ml of cell lysate (mean ± SEM from 6 experiments, unpaired t test). Total protein concentration was 2 mg/ml. (PDF) [file pone.0045993.s005.pdf]
