# Supplementary material for: Differential Regulation of Breast Cancer-Associated Genes by Progesterone Receptor Isoforms PRA and PRB in a New Bi-Inducible Breast Cancer Cell Line
Source: PLoS One. 2012 Sep 24;7(9):e45993. doi: 10.1371/journal.pone.0045993 (PMC3454371; doi:10.1371/journal.pone.0045993)

Figure S6

Differential impact of PRA and PRB expression on molecular pathways. Functional analysis of microarray data was performed using the Ingenuity system database (IPA) for each conditional PR isoform expression. IPA p-values were calculated using right-tailed Fisher's exact test (cut-off : transcriptional FC ±1.3 and p-value <0.001 ; IPA p-value < 0.05). For each pathway and condition, an impact factor (IF) on whole cell transcriptome was calculated as –log(IPA p-value) and represented according to the indicated blue color scaling. Ratio (%) corresponding to the number of PR isoform-dependent genes targeting a given pathway as compared to the total number of known human genes involved in this pathway is represented according to the indicated green color scaling. The indicated IPA pathways impacted by progesterone-independent (vehicle) and/or progesterone-dependent genes (progesterone) are listed according to each conditional PR isoform expression in decreasing order of IF.

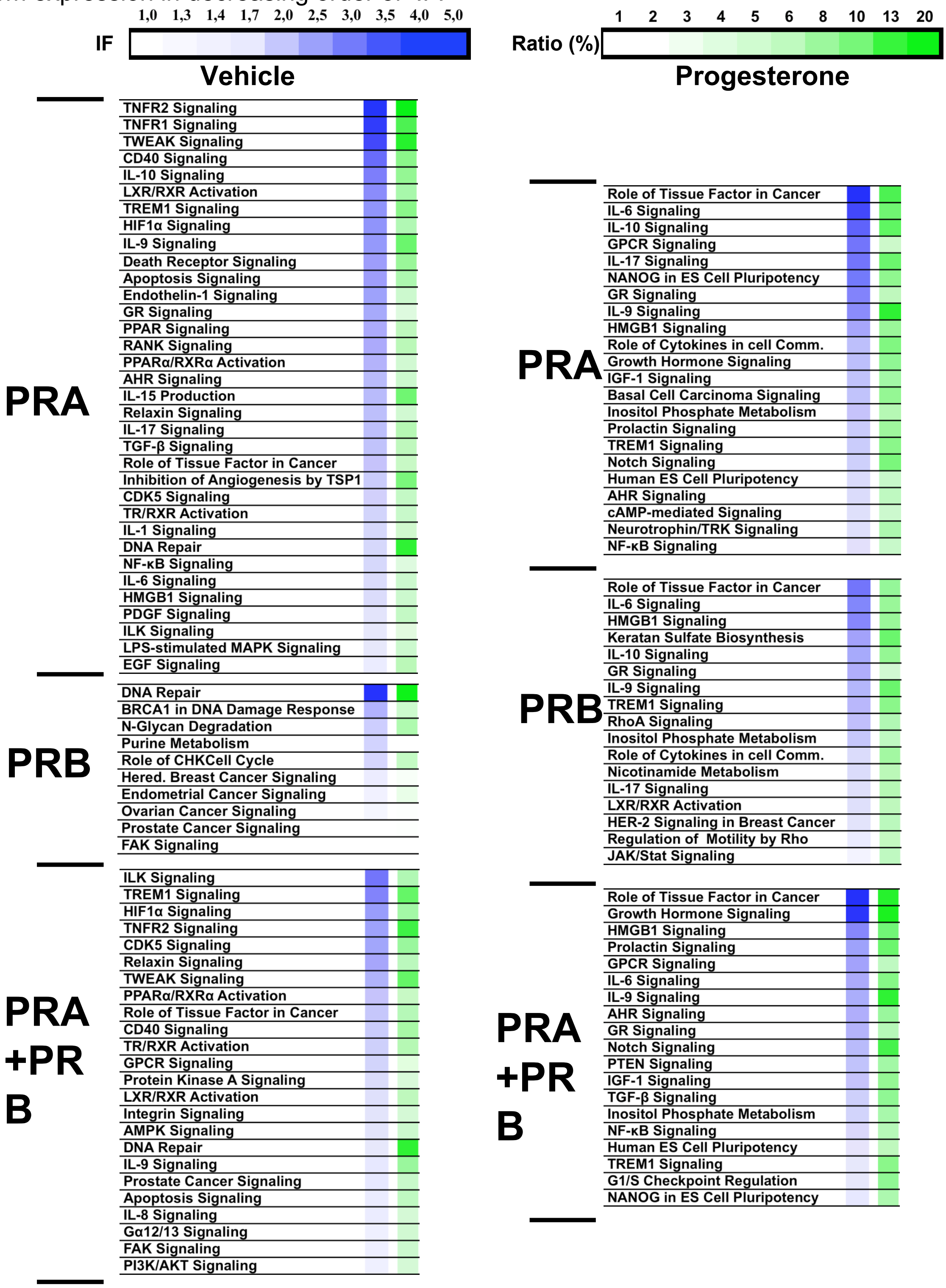

Supplement: Figure S6 — Differential impact of PRA and PRB expression on molecular pathways. Functional analysis of microarray data was performed using the Ingenuity system database (IPA) for each conditional PR isoform expression. IPA p-values were calculated using right-tailed Fisher’s exact test (cut-off : transcriptional FC ±1.3 and p-value <0.001; IPA p-value <0.05). For each pathway and condition, an impact factor (IF) on whole cell transcriptome was calculated as –log(IPA p-value) and represented according to the indicated blue color scaling. Ratio (%) corresponding to the number of PR isoform-dependent genes targeting a given pathway as compared to the total number of known human genes involved in this pathway is represented according to the indicated green color scaling. The indicated IPA pathways impacted by progesterone-independent (vehicle) and/or progesterone-dependent genes (progesterone) are listed according to each conditional PR isoform expression in decreasing order of IF. (PDF) [file pone.0045993.s006.pdf]
