# Supplementary material for: Differential Regulation of Breast Cancer-Associated Genes by Progesterone Receptor Isoforms PRA and PRB in a New Bi-Inducible Breast Cancer Cell Line
Source: PLoS One. 2012 Sep 24;7(9):e45993. doi: 10.1371/journal.pone.0045993 (PMC3454371; doi:10.1371/journal.pone.0045993)

Figure S7

Relative impact of PRA ant PRB on PR-regulated biofunctions. Functional analysis of microarray data was performed using PANTHER system from each conditional expression gene list (A, B, AB) obtained for unliganded (-P4) or liganded (+P4) PR isoforms as described in *Material and Methods*. Following comparison with the whole PR-regulated genes, p-values were obtained using a binomial test, and the most significant functions (p-value <0.01) were extracted. Fraction of genes (%) for each condition participating to a given PR-regulated function targeted by at least 10 genes is mapped on a polar chart (100 % relates to all PR-regulated genes impacting the function irrespective to ligand and isoform expression level). (A) Biological pathways, (B) Cellular processes.

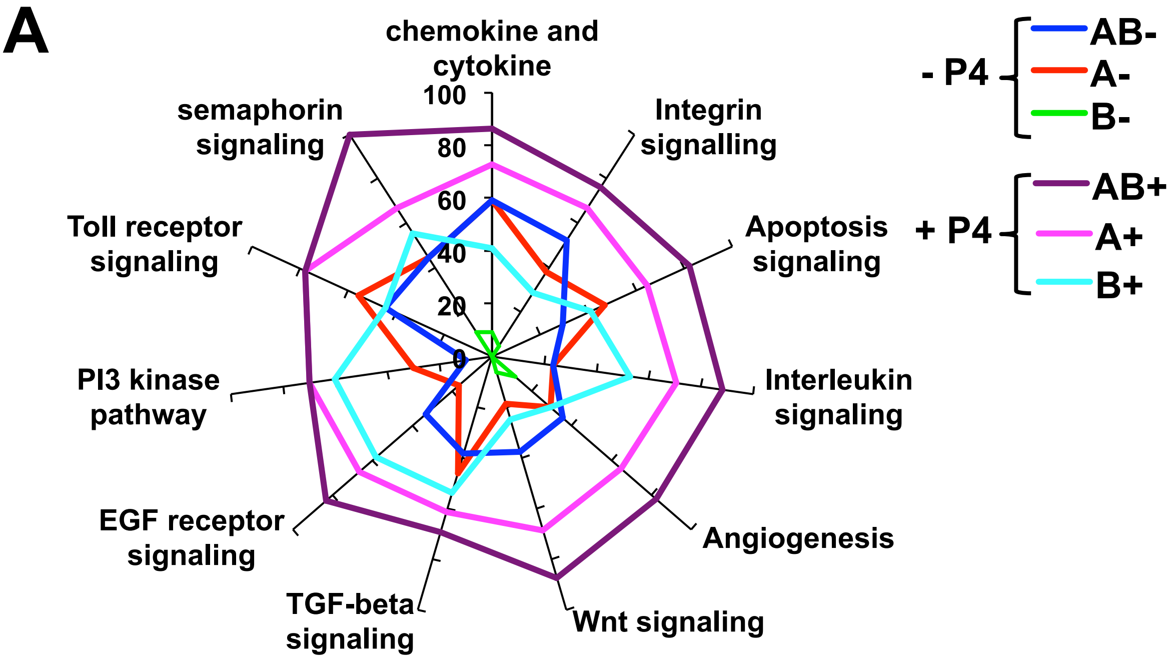

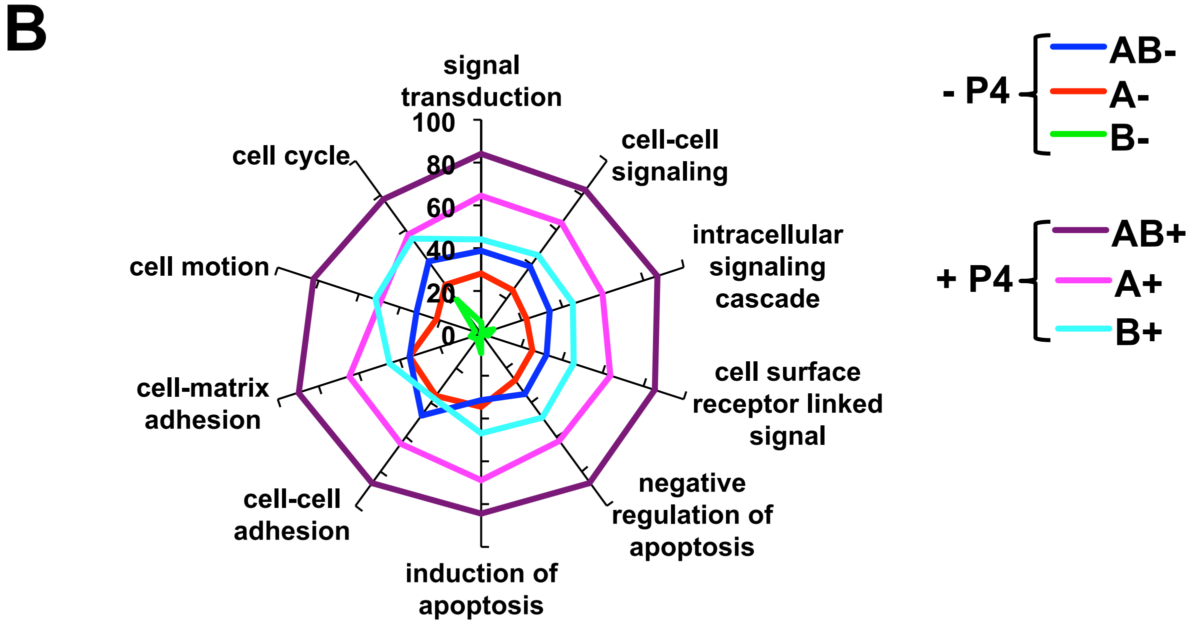

Supplement: Figure S7 — Relative impact of PRA ant PRB on PR-regulated biofunctions. Functional analysis of microarray data was performed using PANTHER system from each conditional expression gene list (A, B, AB) obtained for unliganded (−P4) or liganded (+P4) PR isoforms as described in Material and Methods. Following comparison with the whole PR-regulated genes, p-values were obtained using a binomial test, and the most significant functions (p-value <0.01) were extracted. Fraction of genes (%) for each condition participating to a given PR-regulated function targeted by at least 10 genes is mapped on a polar chart (100% relates to all PR-regulated genes impacting the function irrespective to ligand and isoform expression level). (A) Biological pathways, (B) Cellular processes. (PDF) [file pone.0045993.s007.pdf]
