# Supplementary material for: Differential Regulation of Breast Cancer-Associated Genes by Progesterone Receptor Isoforms PRA and PRB in a New Bi-Inducible Breast Cancer Cell Line
Source: PLoS One. 2012 Sep 24;7(9):e45993. doi: 10.1371/journal.pone.0045993 (PMC3454371; doi:10.1371/journal.pone.0045993)
Supplement: Table S1 — Primer sequences for qPCR experiments. (PDF) [file pone.0045993.s008.pdf]

Supplementary Table S1

Primer sequences used in the study

Primer	Sequence (5’ to 3’)	Gene
FKBP5, Fwd	CCGGAGAACCAAACGGAAA	FK506-binding protein 5
FKBP5, Rev	TGAATGCCACATCTCTGCAGT	
SGK1, Fwd	GTGGCAATTCTCATCGCTTTC	Serum- and glucocorticoid-regulated kinase 1
SGK1, Rev	CTTCAGGGTGTTTGCATGCAT	
HBEGF, Fwd	TGAGCCTCCCAGTGGAAAAT	Heparin-binding EGF-like growth factor
HBEGF, Rev	AACATGAGAAGCCCCACGAT	
AREG, Fwd	ACTCTGGGAAGCGTGAACCAT	Amphiregulin
AREG, Rev	TAGTCATAGTCGGCTCCCGAG	
CCND1, Fwd	ACAGATCATCCGCAAACACG	Cyclin D1
CCND1, Rev	TCTGGAGAGGAAGCGTGTGA	
18S-rRNA, Fwd	GTGCATGGCCGTTCTTAGTTG	18S rRNA
18S-rRNA, Rev	CATGCCAGAGTCTCGTTCGTT	
