# Supplementary material for: Differential Regulation of Breast Cancer-Associated Genes by Progesterone Receptor Isoforms PRA and PRB in a New Bi-Inducible Breast Cancer Cell Line
Source: PLoS One. 2012 Sep 24;7(9):e45993. doi: 10.1371/journal.pone.0045993 (PMC3454371; doi:10.1371/journal.pone.0045993)
Supplement: Table S6 — List of PR-regulated genes impacting cancer. Transcriptional changes induced by expression of PRA and/or PRB in up or down direction that might potentially either decrease (anti) or enhance (pro) tumor growth and/or metastatic evolution. Cancer-related genes were primary selected using IPA system with a cut-off FC ±1.3 and a p-value <10−3. Key genes involved in ‘molecular mechanisms of cancer’ category are indicated (*). The HBEGF and AREG genes studied in the article are underlined. (PDF) [file pone.0045993.s013.pdf]

Supplementary Table S7

Impact of PR-regulated transcription in breast cancer and metastasis

		Ligand-independent			Progesterone-dependent		
		**PRA**	**PRB**	**PRA+PRB**	**PRA**	**PRB**	**PRA+PRB**
**Anti**	**Down**	*ABCC3, BCL3, ,*BIRC3, CXCL1, EDN1, EZR, HBEGF, HES1, ICAM1, ITGAV, JAG1, *JUN, KIAA1199, LAMA5, LMO4, MMP9, *NFKBIE, *NFKB2, PFDN6, RELB, *TGFB2, THBS1*	*KIF20B, PFDN6*	*BCL3, *BIRC3,CSF2, CXCL1, EDN1, FOSL1, HBEGF, ICAM1, ITGAV, JAG1, KIAA1199, MMP9, *NFKBIE, *NFKB2, PDGFRB, PITX2, RELB, *RHOJ, *RHOU, S100P, *TGFB2, THBS1, TMSB4X, TNS4, WNT11*	*ADAM12, ADAMTS1, AGPAT9, ATP6V0A4, BCL2A1, CSF2, CXCL1, CXCR4, CYP1A1, CYP1B1, DDIT4, DKK1, EREG, F2RL1, F3, FOSL1, FRMD4A, *FZD2, HAS2, HBEGF, HDAC9, HRH1, IER2, IL1B, IL6, IL8, INHBA, *IRS1, *JUN, LETM2, LPAR1, *MAP2K6, MLPH, MMP1, MMP3, NAMPT, NR3C2, *PRKCE, *RASGRF1, *RND3, SCG5, SOX9, TFAP2C, UGCG, ZNF367*	*ADAMTS1, AHR, *CDK6, CSF2, CXCL1, CYP1B1, DKK1, EDN1, FOSL1, HAS2, HBEGF, HDAC9, HELB, HES1, HRH1, IER2, IL1B, IL8, INHBA, *IRS1, LETM2, LPAR1, *MAP2K3, MMP1, MMP3, NAMPT, NR3C2, PLK2, *PRKCE, RELB, *RND3, SOX9, TFAP2C, TM4SF1*	*ADAM12, ADAMTS1, AGPAT9, AHR, ATP6V0A4, BCL2A1, *CDK6, CSF2, CXCL1, CXCL2, CXCR4, CYP1A1, CYP1B1, DDIT4, DKK1, EDN1, EMP1, EREG, F3, FOSL1, FRMD4A, *FZD2, HAS2, HBEGF, HDAC9, HELB, HES1, HRH1, IER2, IL1B, IL8, INHBA, *IRS1, ITGA2, *JUN, KRT17, LETM2, LPAR1, MLPH, MMP1, MMP3, NAMPT, NR3C2, PLK2, *PRKAR2A, *PRKCE, *RASGRF1, *RND3, SCG5, SOX9, TFAP2C, *TGFB2, TM4SF1, TNS4, UGCG, ZNF367*
	**Up**	*ADRB2, *ATM, *CASP8, IL1A, MITF, RGS4, SERPINB2*	**ATM, BLID,*	*ADRB2, *ATM, BLID, MITF, PPP2R5C, RGS4, SERPINB2, SMYD4, VHL*	*ADAMTS9, *BMPR2, *CDKN1A, CDKN1C, CEBPD, DACH1, DUSP6, FOXC1, GJB2, HIC1, *JAK1, MT1G, NR4A1, OXTR, PDLIM4, PER1, *PTCH1, SMYD4, TACC2, TFPI2, ZFP36*	*ADAMTS9, *CDKN1A, CDKN1C, CEBPD, CTDSPL, FOXC1, *FOXO1, *JAK1, MT1G, OXTR, PDLIM4, PER1,RGS16, SOCS1, TACC2, ZFP36*	*ADAMTS9, BIK, *CDKN1A, CDKN1C, CEBPD, CTDSPL, DACH1, DUSP6, FOXC1, *FOXO1, GJB2, HIC1, MT1G, NR4A1, OXTR, PDLIM4, PER1, RGS16, SOCS1, SSTR2, TACC2, TFPI2, TNFRSF10B, ZFP36*
**Pro**	**Down**	**JAK1, NR4A1, OXTR, PTX3,*BMPR2*	*BRCA2, RAD50*	*EGLN3, GJB2, NR4A1, OXTR, PTX3, SSTR2*	*ADRB2, BLID, *CASP3, DKK3, IER3, IGFBP1, IGFBP3, IL1A, PTX3, SERPINB2, SOCS3, TNFRSF11B*	**CASP8, IER3, IL1A, PTX3, SERPINB2, SOCS3, TNFRSF11B*	*ADRB2, *ATM, BLID, *CASP3, CLDN1, DKK3, IER3, IGFBP1, IGFBP3, IL1A, MITF, PTX3, SEMA3A, SERPINB2, SOCS2, SOCS3, TNFRSF11B*
	**Up**	*ADAMTS1, AREG, ATP6V0A4, BCL2A11, CA2, CD68, CPA4, DPYSL2, EBLN2, EMP1, FASN, FRMD4A, *GNAS, HSP90AA1, IL1B, KRT17, LOX, MAP1LC3B, MMP1, MMP3, MST1R, ODC1, PPM1J, SEC31B, SLC16A3, ZBTB20*	**CTNNB1, EBLN2, EMP1, FRMD4A, ZBTB20*	*ADAM12, ADAMTS1, AREG, *ARHGEF10, ATP6V0A4, BCAR1, BCL2A11, CA2, CD68, CPA4, *CTNNB1, DPYSL2, EBLN2, EMP1, EPM2AIP1, FASN, FRMD4A, *GNAS, HAS2, HOXB5, HSP90AA1, IL1B, KIDINS220, KRT17, LOX, MALAT1, MAP1LC3B, MMP1, MMP3, MST1R, ORM1, PPM1J, *PRKAR2A, PXN, RBBP6, SEC31B, SLC16A3, ZBTB20*	**BCL2L1, BCL6, COL1A1, CST6, DUSP1, EPOR, EZR, FOSB, FOXA1, *FZD4, *FZD5, ID2, IL6R, IRS2, ITGAV, JAG1, LGR4, LMO4, MMP14, MT1E, MT1H, MT1X, MT2A, PFDN6, *PIK3R3, PITX2, PLAT, *PRKAR2A, PTHLH, SLC19A2, TACSTD2, TNFRSF11A, WNT11, *WNT5A, ZBTB20*	*ADRA1B, AREG, BCL6, *BMP1, CA2, DUSP1, EZR, FHOD3, FKBP5, ID2, IMPDH1, IRS2, LOX, MT1E, MT1H, MT1X, MT2A, MTHFR, PLAT, PTGER4, PTHLH,*RASGRP1, RBBP6, *RHOU, SERPINA1, TNFRSF10C*	*ADRA1B, BCAR1, *BCL2L1, BCL6, COL1A1, CST6, DUSP1, EPOR, FHOD3, FOSB, FOXA1, *FZD4, *FZD5, ID2, IL6R, IRS2, ITGAV, JAG1, LGR4, LOX, MT1E, MT1H, MT1X, MT2A, MTHFR, PDGFRB, PITX2, PLAT, PTGER4, PTHLH, *RHOJ, *RHOU, SDK1, SERPINA1, SLC19A2, TACSTD2, TNFRSF10C, TNFRSF11A, WNT11, *WNT5A*

Transcriptional changes induced by expression of PRA and/or PRB in up or down direction that might potentially either decrease (anti) or enhance (pro) tumor growth and/or metastatic evolution. Cancer-related genes were primary selected using Ingenuity system with a cut-off FC ±1.3 and a p-value < 10^{-3}. Key genes involved in 'molecular mechanisms of cancer' category are indicated (*). The *HBEGF* and *AREG* genes studied in the article are underlined.
